# Accounting for the spread of vaccination behavior to optimize influenza vaccination programs

**DOI:** 10.1371/journal.pone.0252510

**Published:** 2021-06-04

**Authors:** Dor Kahana, Dan Yamin

**Affiliations:** 1 Department of Industrial Engineering, Faculty of Engineering, Tel Aviv University, Tel Aviv, Israel; 2 Center for Combatting Pandemics, Tel Aviv University, Tel Aviv, Israel; East China Normal University, CHINA

## Abstract

Vaccination is the most efficient means of preventing influenza infection and its complications. While previous studies have considered the externalities of vaccination that arise from indirect protection against influenza infection, they have often neglected another key factor–the spread of vaccination behavior among social contacts. We modeled influenza vaccination as a socially contagious process. Our model uses a contact network that we developed based on aggregated and anonymized mobility data from the cellphone devices of ~1.8 million users in Israel. We calibrated the model to high-quality longitudinal data of weekly influenza vaccination uptake and influenza diagnoses over seven years. We demonstrate how a simple coupled-transmission model accurately captures the spatiotemporal patterns of both influenza vaccination uptake and influenza incidence. Taking the identified complex underlying dynamics of these two processes into account, our model determined the optimal timing of influenza vaccination programs. Our simulation shows that in regions where high vaccination coverage is anticipated, vaccination uptake would be more rapid. Thus, our model suggests that vaccination programs should be initiated later in the season, to mitigate the effect of waning immunity from the vaccine. Our simulations further show that optimally timed vaccination programs can substantially reduce disease transmission without increasing vaccination uptake.

## Introduction

Influenza continues to be a significant cause of morbidity and mortality worldwide, imposing substantial health and economic burdens. In the United States alone, there are over 26 million influenza infections annually, with the consequential overall economic burden estimated at $11 billion per year [1]. In Israel, influenza is responsible for over 800,000 infections annually, translating into an economic burden of ~ $261 million per annum [2].

Vaccination is the most efficient and cost-effective way to prevent influenza and its complications [3, 4]. As flu is infectious, vaccination also provides unvaccinated individuals with indirect protection by reducing disease transmission. Thus, it has long been recommended that all individuals above six months of age be vaccinated against seasonal influenza, particularly the elderly and individuals at risk [5, 6]. Nevertheless, vaccination coverage in the vast majority of developed countries, including Israel, is suboptimal [3, 7–10]. Thus, it is essential to improve our understanding of individuals’ vaccination behavior.

It has been established that ideas, information, and behavior can be contagious [11–15]. Survey questionnaire studies showed that advice from coworkers [16], family members or close friends [7, 17] could affect an individual’s decision regarding influenza vaccination. Salathé and Khandelwal suggested that health-related behavior is also reflected in social media, with a strong correlation between the posting of sentiments regarding a new vaccine on Twitter and estimated vaccination rates by region [18]. Therefore, to estimate the predicted vaccination coverage in a population, it is important to take into account that an individual’s vaccination decision depends in part on that of those in his or her social network [19–22].

The transmission of an idea or behavior is similar to the transmission of a biological agent [11, 12]. Ideas and behaviors spread by social contagion, while biological agents spread through biological contagion. Biological contagion, such as the spread of an infectious disease, could be coupled to the social contagion of attitudes related to the disease, such as the utility of vaccination. These two dynamic processes affect one another, and the interplay between them may reveal dynamics that cannot be observed when assessing each process separately [11].

Biological and social contagion can be modeled using a network in which the nodes represent individuals and the links between the nodes represent biological and social connections, respectively. The inherent relationships between infectious diseases and vaccination behavior suggest that they should be examined together [23]. For example, Epstein et al. modeled the coupled spread of an infectious disease and the fear of said disease [24]. Bauch and Bhattacharyya used evolutionary game theory and social learning alongside an epidemiological model to explore the coupled dynamics of infectious disease and vaccination behavior during a vaccine scare [25]. Ellsworth and Salathé modeled the negative sentiments about a vaccine that were spread by complex social contagion and the effect of these sentiments on disease outbreaks [26]. Mao and Yang integrated an epidemiological model to describe disease spread in conjunction with an agent-based adaptive learning model to evaluate the impact of behavior imitation on vaccination coverage [23]. Ruan, Tang and Liu developed an *SIR* with an information-driven vaccination model, whereby the probability of becoming vaccinated in the SIR model describing a disease is determined by an information transmission model [27]. Several studies examined the spread of disease alongside the spread of information about the disease using double-layer multiplex networks [28, 29]. However, to the best of our knowledge, no study has integrated large-scale medical and non-medical data to model the coupled dynamics of vaccination spread and influenza transmission. Our study utilizes real-life locations from high-volume cellular data and is calibrated with longitudinal data from seven influenza seasons that include specific spatio-temporal information on influenza infection and vaccination coverage in Israel.

A comprehensive understanding of the spatiotemporal dynamics of vaccination uptake and its effect on influenza transmission is crucial for improving health policies intended to control the disease. One such policy is the timing of influenza vaccination, which should balance several different considerations. Ideally, vaccination should occur before the onset of influenza activity. However, the temporal dynamics of influenza activity (i.e., the onset, peak, and decline of each influenza season) vary between seasons and are difficult to predict [3]. Furthermore, there is a growing amount of evidence that immunity against influenza wanes over a single season, and a decrease in vaccine effectiveness during a season has been observed [30, 31]. While the peak of influenza activity is usually in January or later [3], the average vaccination coverage through September is 20% of the overall season vaccination coverage in the United States [30]. Individuals who receive vaccination too early may lose their immunity before the end of the influenza season. Therefore, while the waning of the protection conferred by vaccination suggests that vaccination should be delayed, thus ensuring the immunity lasts the entire season, such a delay might result in missed opportunities to vaccinate and in difficulty reaching a high level of vaccination uptake. Accordingly, the U.S. Advisory Committee on Immunization Practices recommends that the timing of influenza vaccination should balance the effects of waning vaccine-induced immunity and the dangers inherent in vaccinating after influenza activity onset [3].

Here, we present a network-based transmission model that simultaneously describes vaccination behavior, a social contagion process, and the propagation of influenza infection in the population. We used a network that we developed from 15 billion records describing the location of ~1.8 million Israeli cellphone users. We calibrated our model to fit high-quality longitudinal data consisting of weekly influenza vaccination uptake and influenza diagnoses over seven years. We further demonstrate how the model can be used to optimize the timing of influenza vaccination to balance these two processes. Our findings show that this real-life network-model captures the spatiotemporal dynamics of vaccination and disease propagation. Moreover, our study underscores the importance of utilizing the coupled process to optimize influenza vaccination timing.

## Results

**Fig 1:**
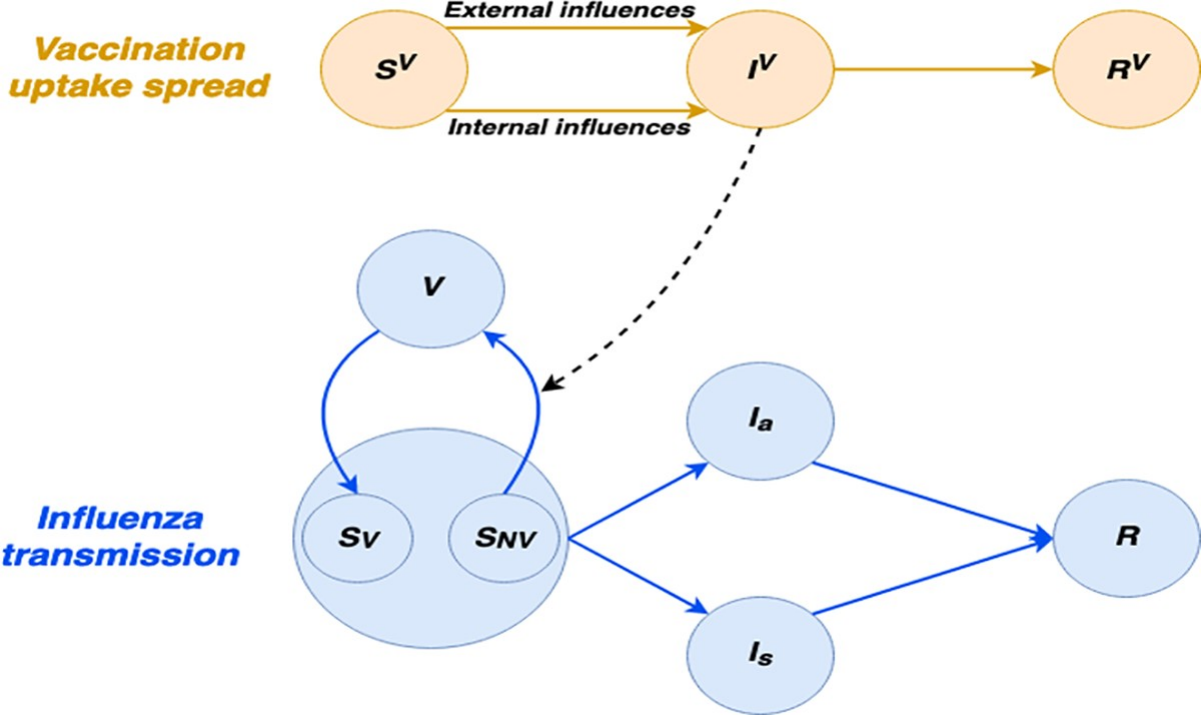
Compartmental diagram of the coupled transmission model. In the vaccination uptake spread component (orange), unvaccinated individuals (S_v_) may either become vaccinated due to an internal decision or an external decision involving the imitation of their contacts who recently got vaccinated (I_v_). Once individuals in the I_v_ class transition to the R_v_ class, they no longer affect their contacts. In the influenza transmission component (blue), susceptible individuals (S_V_ and S_NV_) can move to the infectious class. Susceptible individuals who have not been vaccinated during a given season are labeld S_NV_ and move to the vaccinated class V once they are in the I_v_ in the vaccination uptake spread component. Infectious individuals can be either symptomatic (I_s_) or asymptomatic (I_a_), afterwhich they move to the recovered class (R). Vaccinated individuals can transition back to the susceptible class S_V_ as vaccine immunity wanes during the season. For clarity, the age stratification is not displayed.

We developed a stochastic agent-based model for influenza transmission coupled with vaccination uptake in the population. We modeled the spread of vaccination uptake using a modified Bass-SIR framework [32], wherein unvaccinated individuals could become vaccinated due to an internally motivated decision or an externally motivated decision involving the imitation of their contacts who had just gotten vaccinated. The vaccination uptake model was integrated into a susceptible-vaccinated-infectious-recovered (SVIR) compartmental framework to describe influenza transmission (Fig 1). The lattice for the spread of vaccination uptake and disease transmission is based on a designated contact network we developed using mobility data from the cellphone devices of ~1.8 million users.

**Fig 2:**
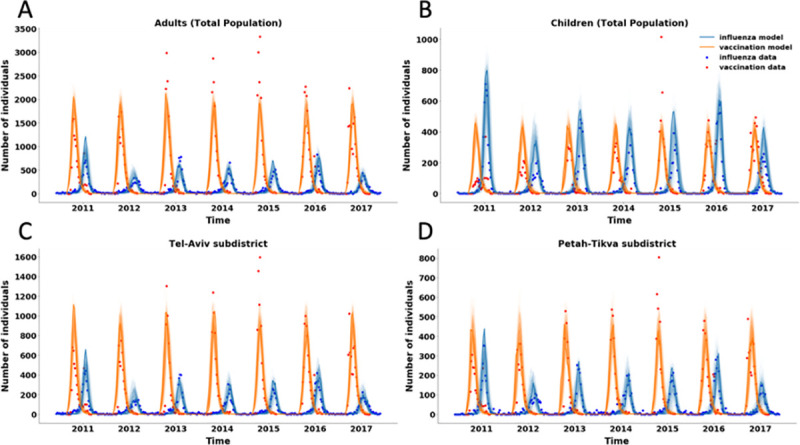
Time series of medical data vs. model fit. Weekly influenza vaccination uptake and influenza cases (dots(, and model fit (lines) between 2010–2017 for children <18 years and adults >18 years (A, B) and in two central subdistricts in Israel (C, D) (see S4 and S5 Figs in the S1 Appendix for the model’s fit for other subdistricts).

We calibrated the model with datasets consisting of weekly vaccination uptake and influenza diagnoses by location and age for seven influenza seasons, between 2010 and 2017. With only three parameters for the vaccination process and two parameters per season for disease transmission, our model simultaneously captures spatiotemporal patterns of both influenza vaccination uptake and disease transmission dynamics (Fig 2A–2D). The model yields a high Pearson correlation fit between the vaccination uptake data (*r* = 0.90) and the influenza diagnosis data (*r* = 0.87).

Our analysis of the human mobility data from cellphones revealed that people of similar demographic groups, such as those with the same religious affiliation and sociodemographic profile, typically travel to zones where the residents match their group. Notably, these trends were not attributable only to geographical distance. Thus, the model with the contact network we developed based on the human mobility data yielded a better fit than a simpler homogenous model in terms of capturing vaccination spread and influenza transmission (S1 Appendix).

**Fig 3:**
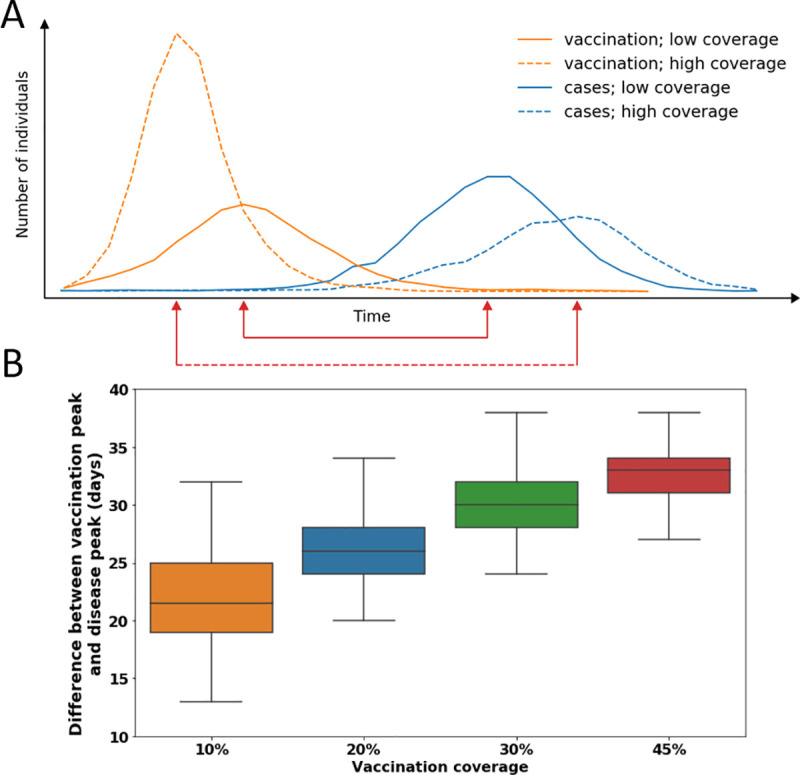
Time difference between vaccination uptake and disease spread. (A) Illustration of the coupled dynamics of the network-based model. Higher overall vaccination coverage leads to more rapid vaccination uptake (dashed vs. solid orange lines) and delays the influenza season (dashed vs. solid blue lines). This prolongs the time between vaccination and disease activity when there is higher vaccination coverage (dashed red line) compared to when there is lower vaccination coverage (solid red line). (B) A boxplot of the time difference between the peak of the vaccination spread and the peak of disease activity for different values of overall vaccination uptake, as observed in our simulations. As illustrated in (A), higher vaccination coverage results in a longer time difference between the peak of the vaccination update and the peak of the disease.

We used the coupled model to study the relationship between vaccination coverage and influenza activity. We found that higher overall vaccination coverage resulted in a more rapid vaccination process. Higher vaccination coverage also reduced the susceptibility to becoming infected in the general population and delayed the onset of influenza. As illustrated in Fig 3A, higher vaccination coverage prolongs the time between vaccination uptake and disease activity. Furthermore, our simulation shows that the higher the overall vaccination coverage, the longer the time between the vaccination peak and the influenza peak (Fig 3B). Given that vaccine-induced immunity wanes over the course of the influenza season, our analysis suggests that vaccination timing should be postponed if higher coverage is anticipated.

**Fig 4:**
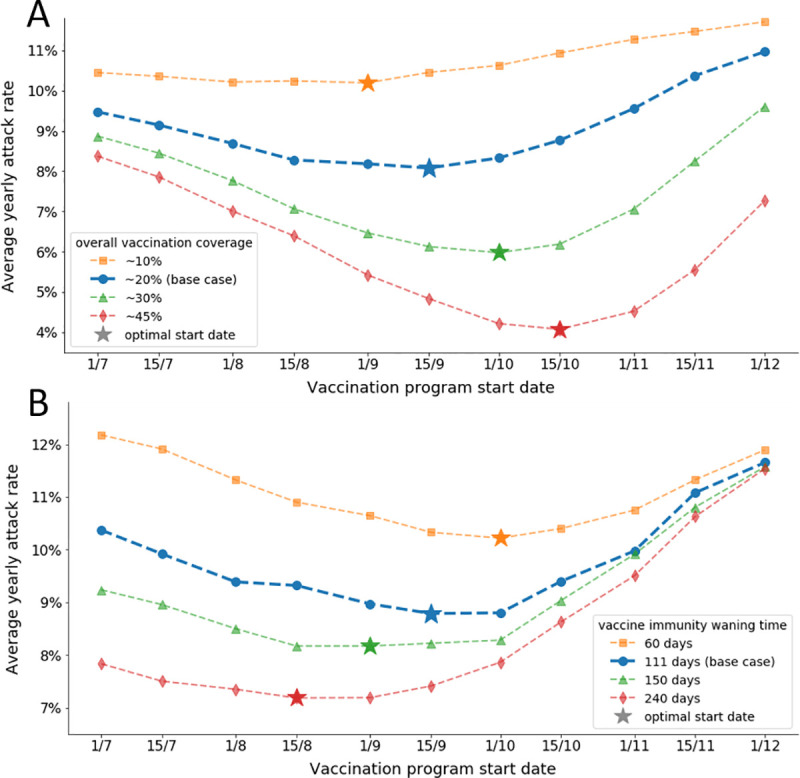
Optimization of the vaccination program start date. Model predictions of the average yearly attack rate over seven seasons for each possible program start date. The star on each line represents the optimal start date for the corresponding line. (A) Optimization for different levels of vaccination coverage. Each line represents a different overall vaccination coverage level, where the bold blue line represents the base case of Israel, i.e., ~20% overall vaccination coverage. (B) Optimization for different values of vaccine-induced immunity waning time, where the bold blue line represents the base case, with a waning time of 111 days.

We utilized the coupled model to optimize vaccination timing for different overall anticipated vaccination coverage levels in Israel. We found that if the total vaccination coverage is 20%, as typically observed in Israel, the optimal timing for vaccination program initiation should be approximately the middle of September. If the coverage level rises to 45%, the vaccination process would faster and, thus, should be initiated in October, to minimize the risk of rapidly waning immunity. Our simulation further indicates that modifying the start date can lead to a substantial reduction in transmission without increasing the vaccination uptake. For example, as our base case suggests, with 20% coverage, shifting the vaccination program’s initiation from October to September would reduce the total attack rate by 7.5% (i.e., from 8.7% to 8.1%). If vaccination coverage were to increase to 45%, shifting the vaccination program’s initiation from September to October would result in a 16.3% reduction in the total attack rate (i.e., from 4.9% to 4.1%) (Fig 4A).

We also performed the vaccination timing optimization process for different values of vaccine waning time. We found that if the time until the vaccine immunity wanes increases, the start date of the vaccination program should be further postponed. For example, for a short pre-waning time of 60 days, the early start date of the vaccination program corresponds to a high average attack rate, as the vaccine immunity declines before the peak of the influenza season. Thus, for this case, a later start date would be more suitable (Fig 4B). On the other hand, if the pre-waning time is long, the intra-season waning immunity is low. Opting for an early vaccination program start date in this scenario would allow more individuals to be vaccinated before influenza season onset (Fig 4B). Finally, our simulations show that if a later start date is chosen for the launch of the vaccination program, a high attack rate is anticipated for all values of the start of immunity waning time, as the time of vaccination spread in the population needs to be taken into account.

## Discussion

Our results indicate that, as vaccination is a socially contagious process, it can be modeled to improve our understanding of influenza transmission. Furthermore, we demonstrate how such improved understanding is beneficial for planning the timing of the initiation of vaccination programs. By modeling the coupling between vaccination spread and disease transmission, we identified complex underlying dynamics that would otherwise have remained hidden. We found that higher vaccination coverage leads to earlier vaccination uptake. As vaccination reduces the susceptibility to becoming infected in the general population, it postpones the start date of influenza activity. Due to the marked rapid waning of vaccine-induced immunity, this coupled dynamic suggests that vaccination programs should be initiated later in regions where coverage is anticipated to be higher. The U.S. Advisory Committee on Immunization Practices recommends balancing the season onset’s unpredictability with waning immunity throughout an influenza season [3]. Our results further suggest that it is essential to also account for the vaccination diffusion process. Taking Israel as a case study, we describe how our model could be used to determine the optimal initiation date of a vaccination program.

We show that a location-based contact network can not only capture disease dynamics but also explain decisions with social contagion aspects. We further demonstrate our model’s ability to capture the coupled dynamics of both vaccination uptake and influenza infection using a contact network we developed from large-scale cellular location data. Typically, studies have addressed the coupled behavior-disease dynamics with a two-layered multiplex network, in which one layer describes the topology relevant from physical contact related to the epidemic [33, 34]. While vaccination decisions may be affected by nonphysical interactions, including mass media [33] and social media [35], we found that the contact network serves as a good proxy for social contagion. The reason for this finding is that people belonging to a certain demographic group, such as those with the same religious affiliation and sociodemographic profile, typically travel to zones in which the residents match their group [36]. Thus, it is likely that their sources of information are similar, which leads to high similarity in the timing of their decision to be vaccinated.

Our work extends previous studies by showing that the decision of whether to be vaccinated or not is consistent across years and that individuals are more likely to be vaccinated if they have been vaccinated in previous seasons [7]. Our study shows that the timing of an individual’s decision to be vaccinated in a season can be explained by social influence. We highlight that our work relies on correlations and associations, as we did not explicitly account for different external factors that could influence influenza vaccination uptake, such as mass media coverage of the disease, healthcare providers’ vaccination campaigns, and vaccine shortages [4, 37, 38]. These factors may result in differences in vaccination uptake between different geographical areas. Nevertheless, as Israel’s healthcare system is centralized, with nationwide vaccination campaigns, there is a lower likelihood of a significant increase or decrease in vaccination uptake in a specific clinic or region.

Given the growing concern regarding childhood vaccine refusal [39], which has recently become a social contagion phenomenon [40], our work is of particular interest. Furthermore, there have been wide-scale campaigns against future SARS-CoV-2 vaccines and wide dissemination online of pro- and anti-vaccine views [41]. We found that people in similar demographic groups, such as those with the same religious affiliation and sociodemographic profile, typically contact each other regardless of their geographical distance, consistent with the findings of other studies [36]. This high degree of clustering of both opinions and contacts suggests that specific populations will be at elevated risk of infection. Therefore, accounting for the spread of behaviors related to vaccination uptake is a significant step towards personalized influenza vaccination campaigns.

The coupled model we developed in this study can also be framed as a multiplex network with two layers, with a asymmetric dynamic interaction between them [29, 42]. In the vaccination layer, the Bass-SIR compartmental framework is used to describe the vaccination spread in the network. In the disease layer, the revised susceptible-vaccinated-infectious-recovered (SVIR) compartmental model is used to describe influenza propagation. The dynamic between the layers is reflected in the transition of nodes from the disease layer to the vaccinated compartment, which is determined by the corresponding nodes in the vaccination layer. Specifically, when a node is infected with the idea of vaccination (i.e., transferred to the *I*^*V*^ compartment in the vaccination layer), its counterpart in the disease layer will be transferred to the vaccinated compartment, where it cannot get infected with the disease.

A comprehensive understanding of the spatiotemporal dynamics of vaccination uptake and its effect on influenza transmission is crucial for improving health policies intended to control the disease. By modeling the decision to become vaccinated against influenza as a socially contagious process, we optimized vaccination program timing. Using our approach, we show how optimally timed vaccination programs can substantially reduce disease transmission without increasing vaccination coverage.

## Materials and methods

We hereby declare that the IRB chair of Tel-Aviv University, Prof. Meir Lahav, determined on March 24, 2020, that there is no need for an IRB approval for this study.

### Model overview

We developed a coupled network-based model for the spread of vaccination uptake and influenza transmission. The model comprises two components: one for vaccination uptake spread and another for disease transmission (Fig 1). The lattice for the spread of vaccination uptake and disease transmission is based on a designated contact network we developed using mobility data from the cellphone devices of ~1.8 million users. For each individual in the network, we track the age group (0–18 years and >18). In addition, at each time step, each individual belongs to a single vaccination-related compartment and infection-related compartment, as specified below.

### Vaccination component

Consistent with previous studies showing that vaccination uptake can be affected by an individual’s social contacts [16], we modeled vaccination spread using methods from studies on product adoption [32]. Specifically, we used the Bass-SIR compartmental framework, wherein during each season, unvaccinated individuals may become vaccinated based on either an internally motivated decision or an externally motivated decision involving the imitation of their contacts who recently were vaccinated. Thus, our compartmental framework stratifies individuals in the network into the three compartments based on their vaccination-adoption status at time *t*: 1) susceptible included unvaccinated individuals (*S*^*V*^), 2) infectious included those who were recently vaccinated and could affect whether their social contacts choose to be vaccinated (*I*^*V*^), and 3) recovered included those who have been vaccinated but are no longer likely to affect whether their contacts choose to be vaccinated (*R*^*V*^). Following the Bass model [43, 44], the probability of vaccination at time interval Δ*t*, that is, the probability of transition from *S*^*V*^ to *I*^*V*^, for an individual *m* is:

$$Prob\left(\begin{array}{c} m\;is\;vaccinated\;in\\ \left(t,t+\Delta t\right) \end{array}\right)=\left(p^{\left(j\right)}+\beta_{v}^{\left(j\right)}\sum_{n\neq m}{a_{nm}\cdot i}_{n}^{V}(t)\right)\Delta t,\;\Delta t\to 0,$$ (1)

where $i_{n}^{V}(t)$ is a state function that gets the value 1 if individual *n* belongs to compartment *I*^*V*^ and 0 otherwise, the parameter *p*^(*j*)^ is the rate of an individual in *S*^*V*^ being vaccinated due to internal, non-social, influences, and $\beta_{v}^{\left(j\right)}$ describes the rate of vaccination due to external, social, influences for age group *j*. The social contacts are given by the components or the adjacency matrix *a*_*mn*_, such that *a*_*mn*_ = 1 if individual *m* is connected to individual *n*, and *a*_*mn*_ = 0 otherwise. Note that as Δ*t*→0, vaccination due to more than one infected contact in a short time interval is negligible.

### Influenza transmission

The compartmental framework [45] stratifies the population into health-related compartments. Specifically, we used a modified susceptible-vaccinated-infectious-recovered (SVIR) model, wherein the states of the disease are classified as susceptible to infection (*S*_*V*_ or *S*_*NV*_), vaccinated (*V*), infected (symptomatic *I*_*s*_ or asymptomatic *I*_*a*_), and recovered (*R*). The *S*_*NV*_ compartment includes non-vaccinated susceptible individuals, while the *S*_*V*_ compartment includes individuals who were immune due to vaccination but are now susceptible due to vaccine-induced immunity wane. We distinguish between these two groups as individuals are not getting vaccinated twice during the course of a season (i.e., no transition from *S*_*V*_ to *V*). Susceptible individuals may contact an infected individual and transition to the infectious compartment, with either asymptomatic or asymptomatic infection. Upon recovery, individuals transition to the recovered compartment. Due to cross-reactive antibodies elicited by previous exposures, we consider an age-specific fraction of each subgroup to be immune at the beginning of each influenza season. This fraction of individuals remain in the immune/recovered compartment for the duration of the influenza season (S1 Appendix). As influenza vaccine efficacy is imperfect, we considered only a proportion of vaccinated individuals to be protected against infection [46]. Susceptible individuals for whom the vaccine was effective, transition from *S*_*NV*_ to the *V* compartment. While an individual is unlikely to be infected with influenza more than once, immunity from influenza vaccination may wane during a single season [30, 31]. Thus, individuals in the *V* compartment may transition to the susceptible compartment *S*_*V*_ (model equations in S1 Appendix).

### Interaction between vaccination and influenza transmission

The coupling of the vaccination model to the influenza transmission model is reflected in the transition from the *S*_*NV*_ compartment to the *V* compartment in the influenza transmission model. This rate is determined by the transitions to the *I*^*V*^ compartment of the vaccination model, as depicted by the dashed black arrow in Fig 1. Essentially, individuals which transition from the *S*^*V*^ compartment to the *I*^*V*^ of the vaccination model (i.e., infected with the idea of vaccination), and belong to the *S*_*NV*_ compartment, will be transitioned to the vaccination of the influenza model (*V*). Specifically, the probability of this transition is determined by the probability of transitioning from *S*^*V*^ to *I*^*V*^ in the vaccination model, as described in Eq (1). Note that to ensure that an individual can not be vaccinated twice during the same season in our model, we explicitly distinguish between susceptible and not vaccinated individuals and those that are susceptible and got vaccinated (i.e., the vaccine was not effective for them or the vaccine immunity waned during the season).

### Force of infection

The probability of a susceptible individual acquiring influenza depends on 1) the contact mixing patterns generated from our contact network, 2) age-dependent susceptibility, 3) the infectiousness of the infectious contact based on the type of infection (i.e., symptomatic or asymptomatic) [47], and 4) seasonal variation. Influenza incidence is seasonal, with a peak typically occurring in the winter, yet the drivers for this seasonality remains uncertain [48]. We thus included general seasonal variation in the force of infection of the model. Taken together, the probability for an individual acquiring an influenza infection from a given contact at time interval Δ*t* is:

$$Prob\left(\begin{array}{c} minfected\;in\\ \left(t,t+\Delta t\right) \end{array}\right)=\left(\Lambda (t)\sum_{n\neq m}a_{nm}\cdot (\rho^{\left(asymp\right)}\cdot i_{a,n}(t)+\rho^{\left(symp\right)}\cdot i_{s,n}(t))\right)\Delta t,\;\Delta t\to 0,$$ (2)

where *ρ*^(*k*)^ is the transmissibility based on the type of infection *k* (symptomatic/asymptomatic), and *i*_*a*,*n*_(*t*) and *i*_*s*,*n*_(*t*) are indicating functions, and are equal to 1 if individual *n* belongs to compartments *I*_*a*_ and *I*_*s*_, respectively, and 0 otherwise. The seasonal forcing rate, *Λ*(*t*) is given by:

$$\Lambda (t)=\beta_{i}\cdot \left(1+cos(\frac{2\pi t}{365}+\phi )\right),$$ (3)

where *β*_*i*_ is the susceptibility rate, and *ϕ* is the seasonality offset (see S2 Table in S1 Appendix).

### Development of the contact network

We generated a contact network based on location and demographic data from various sources. We utilized cellular data from a Radio Network Controller covering central Israel provided by one of Israel’s largest cellular service providers. The data contain 17 billion records describing the location of ~1.8 million anonymized cellphone users over two months. To develop the network, we analyzed approximately 1 million users (accounting for approximately 15 billion records) who had a sufficient number of records and at least one week of cellular data recorded in the dataset.

The data specifies 1) movement patterns within and between 3,070 zones covering Israel, on an hourly basis, from December 2012, until January 2013, 2) the home statistical area for each user inferred by locations identified during nights [49], and 3) age group (<18 years and >18 years) inferred based on proximity to school locations during school hours (S1 Appendix). We integrated these data with demographic data from the Israeli Central Bureau of Statistics (CBS) at the statistical area level [50]. The CBS divides Israel into 3,070 statistical areas, aggregated into cities, sub-districts, and districts. Demographic data are available for each statistical area, including population size, age, ethnicity, and socioeconomic status. We used the CBS data to scale-up the population size to match the Israeli population.

For anonymity, we analyzed only aggregated data that details in each hour the number of individuals that their home is in statistical area *i*, and that are in statistical area *j* and belong to age group *l*. More specifically, a *visit distribution matrix* was calculated based on the location distribution. In this matrix, each row represented the home statistical area and age group of individuals, and each column represented the proportion of time they spent in each statistical area. An *attendance matrix* was generated to describe the proportion of individuals from area *i* and age group *j* in each of the statistical areas. We defined contact in a probabilistic manner, i.e., as the probability that an individual from statistical area *i*, in age group *j*, will contact an individual from statistical area *k*, in age group *l*. The element *P*_(*i*,*j*),(*k*,*l*)_ of the *contact probability matrix* is:

$$P_{\left(i,j),(k,l\right)}=\sum_{n=1}^{N}V_{(i,j),n}\cdot V_{(k,l),n}\cdot A_{(k,l),n},$$ (4)

where *N* is the number of statistical areas, *V* is the *visit distribution matrix*, and *A* is the *attendance matrix*. Essentially, this probability is a summation over all the statistical areas of the probability that both individuals will visit area *n*, multiplied by the proportion of individuals from area *k*, in age group *l* attending area *n*. We normalized each row to a sum of one to determine the conditional probability that an individual from area *i*, in age group *j* will contact an individual from area *k*, in age group *l*, given that a contact occurred. The resulting matrix is the *conditional contact probability matrix* (see S2 Fig in S1 Appendix).

To examine interactions between geographical areas, the *conditional contact probability matrix* was aggregated by subdistricts (S2A Fig in S1 Appendix). To examine interactions between different socioeconomic groups, we aggregated the matrix by socioeconomic score affiliated with each statistical area, based on the CBS socioeconomic data (S2B Fig in S1 Appendix). The socioeconomic score is measured on a scale from 1 to 20, where 1 indicates the lowest socioeconomic status, and 20 indicates the highest socioeconomic status (aggregation by ethnic group is provided in the S1 Table in S1 Appendix).

We generated a simulation-based contact network with 100,000 nodes. The network is represented by an undirected graph in which each node represents an individual, and the edges represent contacts between individuals. The statistical area and age group distribution of the nodes are based on the CBS demographic data. The number of contacts for each node is generated independently from a geometric distribution, with a different average for each age group derived from contact mixing surveys [51, 52]. The contact distribution for each node (i.e., the number of contacts with individuals from each statistical area and each age group) was generated based on the node’s home statistical area and age group and the corresponding row of the *conditional contact probability matrix*. The edges between nodes were connected randomly under these constraints (S1 Appendix).

### Model calibration

The model includes three free parameters for the vaccination component and two free parameters per season for the disease component (S3 Table in S1 Appendix). To estimate these unknown parameters, we calibrated the model to weekly vaccination uptake data and influenza and influenza-like-illness (ILI) diagnoses by age and geographic location. These data were collected by the Maccabi Health Maintenance Organization (HMO), the second-largest HMO in Israel [53]. The queried electronic medical records include longitudinal data for 250,000 members (randomly assigned) between 2010 and 2017. For each patient, a wealth of information is available, including demographic characteristics, the diagnosis of respiratory infections, and vaccination uptake. To account for unreported cases, the weekly influenza diagnosis data were adjusted to fit actual attack rates by age group reported in previous studies in Israel [2].

We assumed that the number of newly vaccinated individuals each week and the number of newly infected individuals each week are independent variables. Given this assumption, we used the Poisson distribution to describe these variables separately for each subdistrict and age group pair. We then chose the set of parameters that maximizes the following likelihood function:

$$L(\theta )=\prod_{s,j,w}\frac{e^{-{\hat{\lambda }}_{sjw}}\cdot {\left({\hat{\lambda }}_{sjw}\right)}^{d_{sjw}}}{d_{sjw}!},$$ (5)

where *θ* is the set of parameters of the vaccination/disease component, *d*_*sjw*_ represents the weekly data (i.e., the weekly vaccination uptake/influenza and ILI diagnoses for subdistrict *s*, age group *j* and week *w*), and ${\hat{\lambda }}_{sjw}$ is the Poisson distribution parameter estimated from the model for each subdistrict *s*, age group *j*, and week w (S1 Appendix). We applied the Akaike information criterion, derived from information theory, to compare the network-based model to a homogenous model [54]. To compare the models, the homogenous model is based on the same network with all nodes connected to each other. The probability of contact between nodes differs by age (S1 Appendix).

### Optimization of vaccination program timing

To optimize the timing of vaccination programs, we examined different program start dates ranging from July 1 to December 1 in half-month steps. We used the coupled transmission model to simulate the vaccination and disease propagation over the seven seasons for each of the program start dates (S1 Appendix). We evaluated the average yearly attack rate across all the simulations and the seven seasons. We then compared the attack rates among the different model settings to find the optimal vaccination program start date, representing the start date that corresponded to the lowest average yearly attack rate. Altogether, the optimization process included 30,800 model iterations (4 vaccination coverage values, 11 vaccination programs start dates, 7 seasons, and 100 iterations per setting). Each iteration of the coupled model takes on average 25.4 seconds ± 300 ms, using Intel Core i9-7920X CPU (S1 Appendix).

We used the calibrated model to simulate a specific season with different values of overall vaccination coverage. We then calculated the peak of the vaccination process, the peak of influenza activity, and the distance between them (Fig 3A). We also performed this analysis for different values of overall anticipated vaccination coverage (10, 20, 30, and 45%) and examined the time difference for each setting (Fig 3B). Furthermore, we performed a sensitivity analysis of overall vaccination coverage by optimizing the vaccination program start date for four different levels of vaccination coverage (10, 20, 30, and 45%) and comparing the optimal dates (Fig 4A). Different values of overall vaccination coverage were obtained by modifying the infection probability parameters of the vaccination component (S7 Table in S1 Appendix). Finally, we performed a second sensitivity analysis, examining the impact of different values of vaccine-induced immunity waning time by optimizing the vaccination program start date for four different levels of vaccination waning time (60, 111, 150, and 240 days) and comparing the optimal dates (Fig 4B).

## Supporting information

S1 Appendix Supporting information for accounting for the spread of vaccination behavior to optimize influenza vaccination programs. (DOCX)
