# Supplementary material for: Accounting for the spread of vaccination behavior to optimize influenza vaccination programs
Source: PLoS One. 2021 Jun 4;16(6):e0252510. doi: 10.1371/journal.pone.0252510 (PMC8177529; doi:10.1371/journal.pone.0252510)
Supplement: S1 Appendix — (DOCX) [file pone.0252510.s001.docx]

Supplementary Information for: Accounting for the spread of vaccination behavior to optimize influenza vaccination programs

Dor Kahana^1^, Dan Yamin^1^

1 Department of Industrial Engineering, Faculty of Engineering, Tel Aviv University, Tel Aviv 69978, Israel

**Content**

[1. Network development 2](#_Toc68018498)

[1.1. The Data 2](#_Toc68018499)

[1.3. Data Preprocessing 3](#_Toc68018500)

[1.4. Contact probability matrix development 3](#_Toc68018501)

[1.5. Network generation 8](#_Toc68018502)

[2. Transmission model development 10](#_Toc68018503)

[2.1. Model transitioning 11](#_Toc68018504)

[2.3. The Seasonal rate 13](#_Toc68018505)

[2.4. Model parameters 14](#_Toc68018506)

[2.5. Model calibration 15](#_Toc68018507)

[2.6. Comparison with a homogenous model 19](#_Toc68018508)

[3. Optimization of influenza vaccination program 20](#_Toc68018509)

[3.1. Base case 20](#_Toc68018510)

[3.2. Sensitivity analysis 21](#_Toc68018511)

[4. Network model pseudocode and complexity 22](#_Toc68018512)

[4.2. Vaccination component 22](#_Toc68018513)

[4.3. Disease component 24](#_Toc68018514)

[4.4. Complexity 26](#_Toc68018515)

# Network development

## The Data

We utilized data from various sources:

### Cellular location data

This dataset includes cellular data from Radio Network Controller (RNC) covering central Israel, provided by one of Israel's largest cellular service providers. The cellular data contain 16,826,157,036 records describing the location of 1,799,977 cellphone users over two months, between December 2012 and January 2013. The data is anonymized, i.e., users' International Mobile Subscriber Identifier (IMSI) have been randomly assigned and aggregated based on the following decision rules:

- **Home statistical area** – the data were aggregated according to 3,070 home statistical area according to a specified algorithm for identification of the home statistical area.
- **Age Stratification** – the users were aggregated to two age groups: 0-18 years and >18 years.

### Central Bureau of Statistics data

We used demographic and socioeconomic data from the Israeli Central Bureau of Statistics (CBS). The CBS divides Israel into 3,070 statistical areas, that are aggregated into 1,235 cities, 15 subdistricts, and 7 districts. Demographic and socioeconomic data are available for each statistical area. The cellular data covers central Israel and includes 1,804 statistical areas, 569 cities, 10 subdistricts, and 6 districts. The available data include:

- **Location** – a polygon object for each statistical area, indicating its borders. All statistical areas are disjoint.
- **Demographic data** – based on the 2008 census, the data is available for 2,299 statistical areas and includes population size by age (0-17, 18-64, 65+), main religion, and percentage of main religion.
- **Socioeconomic status** – For each statistical area, a socioeconomic score is available. The score is calculated by the CBS using factor analysis and cluster analysis based on different factors such as demographics, education, employment characteristics, and unemployment level. Based on this analysis, statistical areas are affiliated to one of 20 clusters, where 1 indicates the lowest socioeconomic status and 20 indicated the highest socioeconomic status. The data is available for 1,866 statistical areas.

### Survey of Israel data

We used location data of educational institutions from the Survey of Israel. The data contain the location of the institute (longitude and latitude), a category (kindergarten, school, university), and a subcategory (for school – elementary school, middle school, high school, and religious school).

## Data Preprocessing

We removed users with fewer than 50 records or less than one week recorded in the data. After removing these users, the data contain 985,997 users (14,903,823,478 records). Likewise, we added a *statistical area id* for each record in the cellular location data, corresponding to the location of the call. Namely, for each record, we found statistical area polygon containing the corresponding point, described by the couple $\left( longitude,latitude \right)$.

## Contact probability matrix development

### Identifying a home statistical area

To aggregate users by statistical areas, we defined a home statistical area for each user in the cellular data. In line with previous studies involving analysis of cellular data (1), the home location of an individual was inferred based on her location distribution by statistical area during the night.

For each user, we calculated the proportion of records from each statistical area he visited during the night, specifically between 8 PM and 8 AM. Then, we used the following statistical hypothesis testing. Denote the two largest proportions for each user by $\hat{p}_{I},\hat{p}_{II}$:

$$H_{0}:p_{I}=p_{II}$$

$$H_{1}:p_{I}>p_{II}$$

Specifically, if $H_{1}$ is accepted, i.e., $p_{I}>p_{II}$, the area corresponding to $p_{I}$ is determined to be the home statistical area. Otherwise, the home statistical area is not determined.

We used simulations for hypothesis testing. We generated 10,000 values from a multinomial distribution, under $H_{0}$:

|  | $X Multinomial\left( n,p,p,1-2p \right),$ | (1) |
| --- | --- | --- |

where $p=\frac{\hat{p}_{I}+\hat{p}_{II}}{2}$ and $n$ is the total number of records during the night for the current user. We then determined with a significance level of 25% whether $H_{0}$ is rejected.

Based on this approach, we were able to define a home statistical area for 80% of the users in the cellular data.

### Age Stratification

We divided the users in the cellular data into two age groups: 0-18 years and >18 years. Because the users in the cellular data are anonymized, we used school location data to infer an individual's age group based on her proximity to a school. For each user, we calculated the proportion of records indicating a location within 200 meters from a school between 9 AM and 11 AM on weekdays. If this proportion exceeds a threshold of 0.5, we determine the age group of the individual to be 0-18. Otherwise, the age group is determined to be >18.

To validate these results, we examined measures of distance-traveled for each age group (Fig. S1), under the assumption that children travel shorter distances compared to adults. Our results indicate that individuals from the 0-18 age group travel distances, deduced by the number of areas visited and radius from home, are shorter on average compared to individuals from the >18 age group.

| 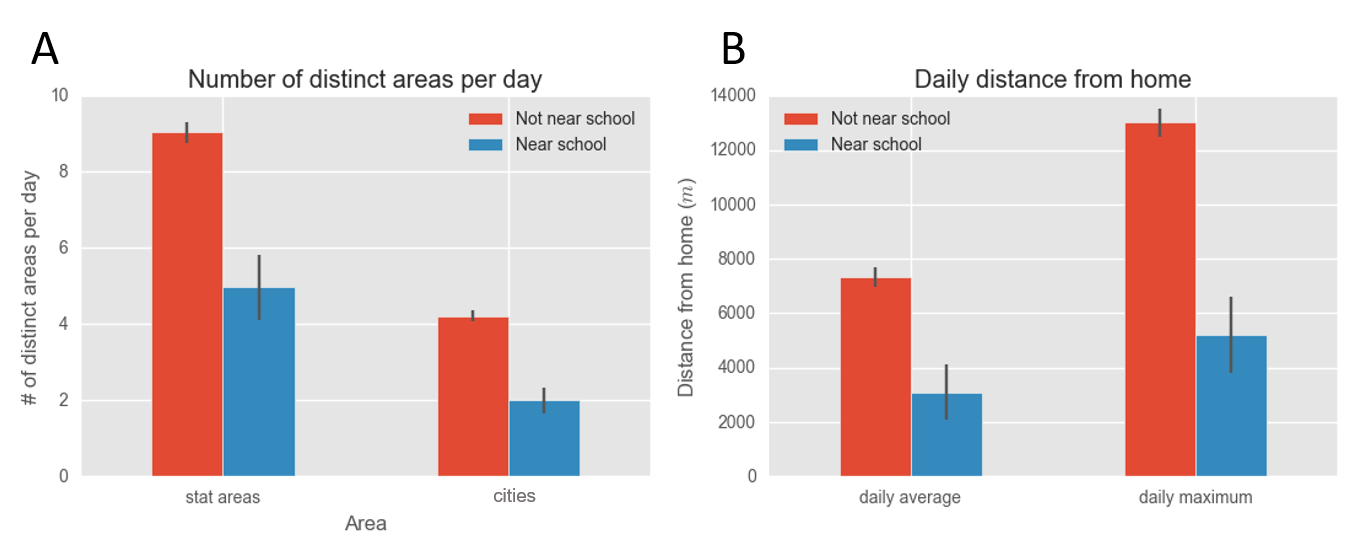 |
| --- |
| **Fig. S1.** Distance-traveled measurements by proximity to a school. The red bars represent individuals who belong to the 0-18 age group, and the blue bars represent individuals who belong to the >18 age group based on 1.3.2.). (A) The number of distinct statistical areas and cites visited per day. (B) The daily average and maximum distance from the center of the home statistical area. |

### Defining a contact

We defined a contact in a probabilistic manner as the probability that an individual from statistical area $i$, age group $j$, contacts an individual from statistical area $k$, age group $l$. The element $P_{\left( i,j \right),\left( k,l \right)}$ of the *contact probability matrix*, is:

|  | $P_{\left( i,j \right),\left( k,l \right)}=\sum_{n=1}^{N} V_{\left( i,j \right),n}\cdot V_{\left( k,l \right),n}\cdot A_{\left( k,l \right),n},$ | (2) |
| --- | --- | --- |

where $N$ is the number of statistical areas, $V_{\left( i,j \right),n}$ is the probability that an individual from area $i$ age group $j$ visits area $n$, and $A_{\left( k,l \right),n}$ is the proportion of individuals from area $k$, age group $l$, attending area $n$ (out of all individuals attending area $n$). Essentially, this probability is a summation over all the statistical areas of the probability that both individuals visits area $n$, multiplied by the proportion of individuals from area $k$, age group $l$, attending area $n$.

### Visit distribution matrix

The *visit distribution matrix* $V$ ($2N\times N$) describes the visit distribution of an individual from area $i$, age group $j$ in each of the $N$ statistical areas. In this matrix, each row represents a home statistical area and age group of individuals, and each column represents the proportion of time they spend in each statistical area (the rows of the matrix sum to 1). The matrix was calculated based on location distribution during active hours, i.e., between 6 AM to 11 PM. For each home statistical area $i$ and age group $j$ we calculate the average proportion of records from each statistical area over all users from statistical area $i$ age group $j$.

### Attendance matrix

The *attendance matrix* $A_{T}$ ($2N\times N$) describes the proportion of individuals from area $i$ age group $j$ in each of the $N$ statistical areas (The columns of the matrix sum to 1). The matrix was calculated based on the *visit distribution matrix* $V$. To account for oversampling and under-sampling of statistical areas in the cellular data, each row of the visit distribution matrix was multiplied by the corresponding population size based on the CBS data. Now, the matrix represents the average number of individuals from each statistical area and age group attending each of the statistical areas. Then, we normalized each column to a sum of one to receive the proportion of individuals from each statistical area and age group in each of the statistical areas.

### Contact probability matrix

After calculating the *visit distribution matrix* and the *attendance matrix*, we calculated each element of the *contact probability matrix* according to formula [1]. The contact probability matrix describes the probability that an individual from statistical area $i$ and age group $j$ has a contact with an individual from area $k$ and age group $l$.

Statistical areas that are not included in the cellular data, as well as couples of $\left( statisticalarea,agegroup \right)$ that none of the users in the cellular data were affiliated to, were removed in the process of calculating the contact probability matrix. Therefore, the dimensions of the final *contact probability matrix* are $\left( 2049,2049 \right)$.

Finally, we normalized each row of the *contact probability matrix* to a sum of 1 to obtain the *conditional contact probability matrix*, which describes the probability that an individual from statistical area $i$ and age group $j$ has a contact with an individual from area $k$ and age group $l$, given that a contact occurred.

### Aggregation of the contact probability matrix

To calculate the *conditional contact probability matrix* in *city-level* and *subdistrict-level* (Fig. S2A), we aggregated the *visit distribution matrix* by city and subdistrict, respectively, and repeated the process described in 1.3.5-1.3.6. Also, we calculated the socioeconomic contact probability matrix (Fig. S2B) by aggregating the *visit distribution matrix* by the socioeconomic score affiliated to each statistical area according to the CBS data. The religious-group contact probability matrix was calculated by aggregating the statistical areas based on the religion affiliated with the statistical area according to the CBS demographic data (Table S1).

| 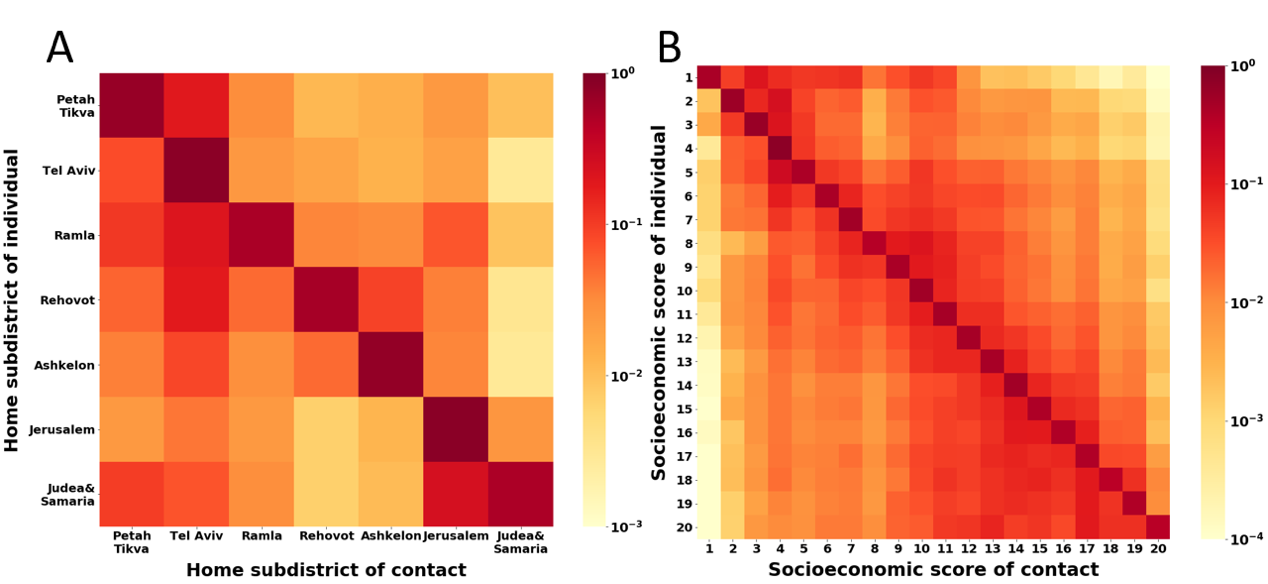 |
| --- |
| **Fig. S2.** Contact probability heat maps. Each row identifies an *individual's* home subdistrict (A) or socioeconomic score (B). Each column identifies the *contact's* home subdistrict (A) or socioeconomic score (B). The color at the intersection of the row and column indicates the conditional probability of a contact between the two, given that a contact occurred. The heat maps are aggregations of the statistical-area-level contact matrix (*Methods*). (A) An aggregation by geographic location, i.e., subdistrict. (B) An aggregation by the socioeconomic score of each statistical area on a scale of 1-20, where 1 indicates the lowest socioeconomic status and 20 indicates the highest socioeconomic status. |

| **Table S1.** Religious-group contact matrix. Each row identifies an *individual's* religion, and each column identifies the *contact's* religion. The number at the intersection of the row and column indicates the conditional probability of a contact between the two, given that a contact occurred. The heat map is an aggregation of the statistical-area-level contact matrix by religion. | | |
| --- | --- | --- |
|  | **Jewish** | **Muslim** |
| **Jewish** | 0.96 | 0.04 |
| **Muslim** | 0.54 | 0.46 |

### Area centrality

To assess the centrality of areas, we calculated the PageRank of each area (2, 3), using the contact probability matrix as the adjacency matrix. We used a damping factor of 1, i.e., transitions are made only according to the adjacency matrix. The PageRank values in a subdistrict level are described in Fig. S2 in descending order. The two most central subdistricts are Tel Aviv (with PageRank of 0.45) and Jerusalem (with PageRank of 0.19). However, if we account for the population size by dividing the PageRank by the relative population size of each subdistrict out of the total population size, the two subdistricts with the highest centrality score are Tel Aviv and Petah Tikva.

| 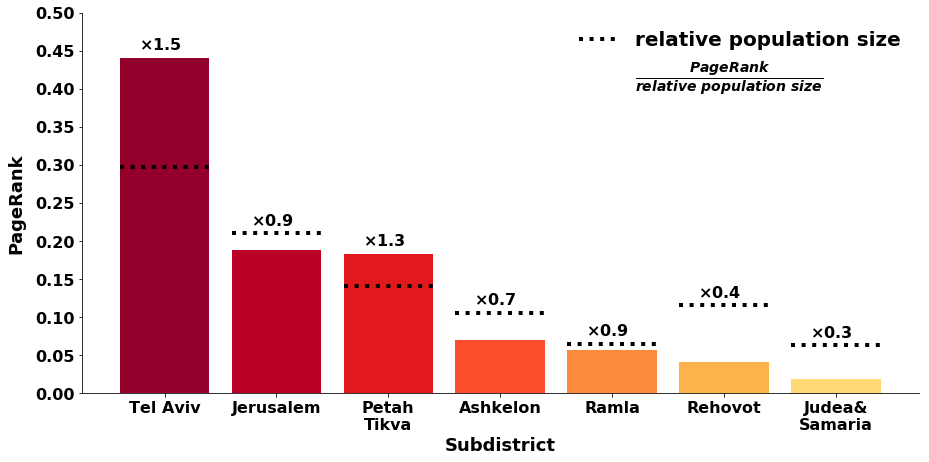 |
| --- |
| **Fig. S3.** Area centrality. The bars represent the PageRank score of each subdistrict, the dotted line represents the relative population size of each subdistrict out of the total population size of all seven subdistricts, and the number at the top of each bar is the Page rank divided by the relative population size. |

### Using the contact matrix for the vaccination process

Based on the vaccination coverage data described in 2.5.1, we show that geographic patterns, as well as socioeconomic patterns, are correlated with vaccination decisions (Fig. S3). Specifically, the vaccination coverage differs by geographical area (Fig. S3 A). Likewise, the higher the socioeconomic score, the higher the average vaccination coverage (Fig. S3 B). These patterns intensify the notion of modeling vaccination uptake as a social contagion process.

| 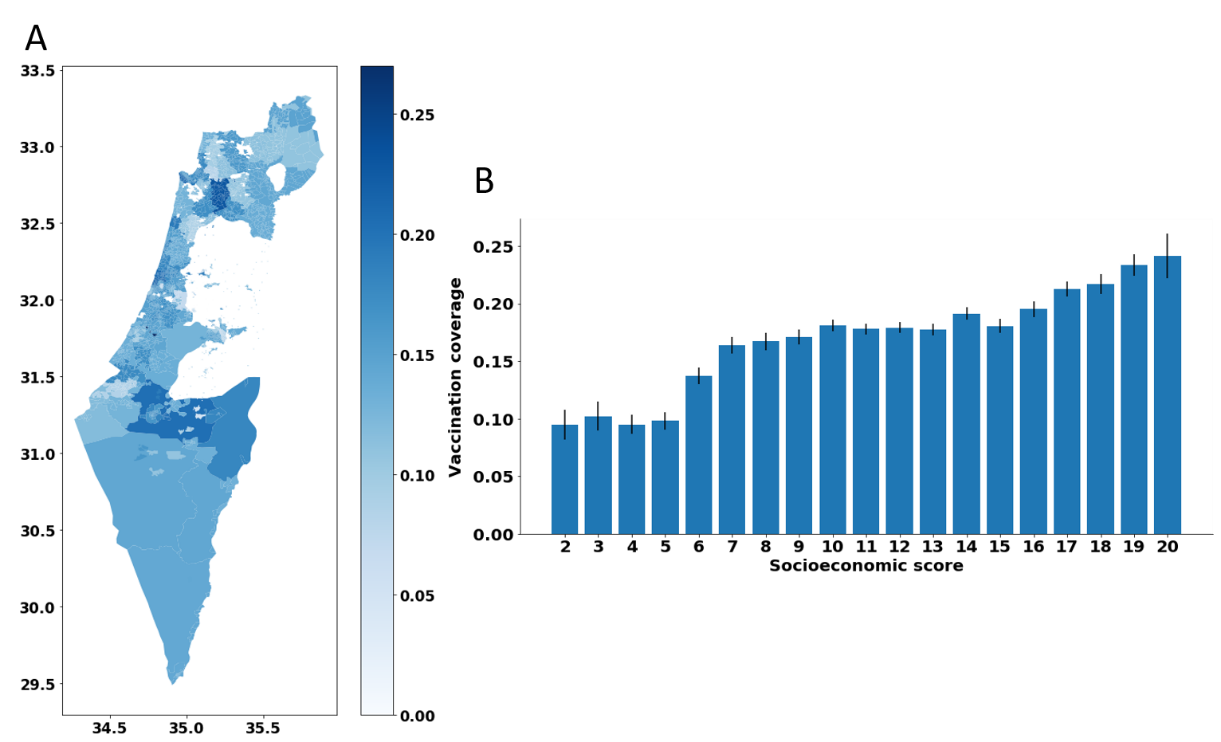 |
| --- |
| **Fig. S4.** Vaccination coverage by geographical area and by socioeconomic score. (A) Vaccination coverage by geographical area. The color indicates the average vaccination coverage in each area, the darker the color, the higher the vaccination coverage. (B) Average vaccination coverage by socioeconomic score. |

## Network generation

### Initialization

We generated a simulation-based contact network with 100,000 nodes. The network is represented by an undirected graph $G=\left( N,E \right)$, where each node represents an individual, and edges represent contacts between individuals. We denote the adjacency matrix $A$ of size $N\times N$, such that $a_{mn}=1$ if individual $m$ is connected to individual $n,$ and $a_{mn}=0$ otherwise. Each node is assigned to an age group and a statistical area out of the couples of $\left( statisticalarea,agegroup \right)$ that are available in the *contact probability matrix*. The number of nodes from each statistical area and age group is determined according to the relative population size based on the CBS demographic data.

### Distribution of the daily number of Contacts

Assuming the independence of contacts, we generated the daily number of contacts from a geometric distribution, with a different average for each age group. The average number of contacts for each age group was derived from an age-group-contact-matrix based on surveys conducted in Israel and 151 more countries (4). The survey contact matrix specifies the total number of contacts per day by age groups of 5 years. We used the CBS demographic data to calculate the weighted average number of contacts for the two age groups of the network (0-18 and >18). The resulted average number of contacts per day for the 0-18 age group is 16.83, and for the >18 age group is 12.14.

The contact distribution for each node, i.e., the number of contacts with individuals from each statistical area and each age group, was generated based on the node's home statistical area and age group, and the corresponding row of the *conditional contact probability matrix*. Specifically, the number of contacts from each statistical area and age group was generated from the following multinomial distribution:

|  | $X Multinomial\left( n,p_{1},p_{2},\ldots,p_{N} \right),$ | (3) |
| --- | --- | --- |

where $n$ is the number of contacts and $p_{1},\ldots,p_{N}$ is the row in the *conditional contact probability matrix* corresponding to the statistical area and age group of the individual. We denote this contact distribution as the *planned-contact-distribution*, which specifies the number of required contacts from each couple of *(statistical area, age group).*

We define a couple of *(statistical area, age group)* as a sparse if it includes fewer than 30 users in the cellular data (1,290 out of 2,049 couples). For these couples of *(statistical area, age group)*, we use the contact distribution in the city-level, due to the small sample size. Specifically, we use the distribution from equation [3] where $p_{1},\ldots,p_{N}$ is the row of the *city-level* *conditional contact probability matrix* corresponding to the individual's home city. The statistical area within the resulting cities is chosen randomly.

### Connecting between nodes

We define the *remaining-contact-distribution* as the contacts out of the *planned-contact-distribution* that does not yet exist as edges in the graph. Initially, the *remaining-contact-distribution* is identical to the *planned-contact-distribution*.

Edges between nodes are connected under the contact distribution constraints. Namely, we go over the nodes iteratively and create edges based on the origin node's contact distribution and the contact distributions of the destination nodes. We go over the nodes in two phases. In the first phase, for each origin node, we examine the *remaining-contact-distribution*, and for each of the couples *(statistical area, age group)* with a number of contacts greater than zero, we define a list of candidates. The candidates are nodes from the relevant statistical area and age group, which have a positive number of contacts with nodes from the statistical area and age group of the origin node in their *remaining-contact-distribution*. The destination nodes are chosen randomly from the candidate list, and the *remaining-contact-distribution* of both origin and destination nodes is updated, respectively. Whenever a given contact does not have available candidates, we skip it. After the first phase, 50% of the planned contacts exist as edges in the network. The *remaining-contact-distribution* of each node now includes contacts that could not be created, if any, as there were no relevant candidates. To create more of the planned contacts, in the second phase, we go over the nodes again and create the list of candidates in a subdistrict level. Namely, the candidates are now nodes from the relevant subdistrict and age group, which have a positive number of contacts with nodes from the subdistrict and age group of the origin node in their *remaining-contact-distribution*. This way, more relevant candidates could come up, and more contacts can be created. After the second phase, more than 95% of the planned contacts exist as edges in the network. We ignore the remaining 5% of contacts that do not exist as edges and remove from the network nodes with a degree of zero.

# Transmission model development

We developed a coupled network-based dynamic model for both influenza vaccination and influenza infection transmission in Israel. Our model is a modified susceptible-infected-recovered (SIR) compartmental framework (5) in which transitions between the classes occur over time. The model comprises two components, one for the vaccination transmission and another for the disease transmission (Main text, Fig. 1). The season length for the disease component is 365 days, between June and May of the following year. For the vaccination component, the season length is 180 days, in the base case between September and February the following year. At each time step, i.e., one day, the state of each node in the network is defined by its association to one class of the vaccination component, and one class of the disease component.

Accordingly, we stratified the population into three vaccination-related classes and five disease-related classes. The vaccination-related classes are regarding the decision to become vaccinated and include: susceptible $S^{V}\left( t \right)$, infectious $I^{V}\left( t \right)$ and recovered $R^{V}\left( t \right)$. The disease-related classes are: susceptible who did not get vaccinated during the current season $S_{NV}\left( t \right)$, susceptible who got vaccinated but their vaccination waned $S_{NV}\left( t \right)$, vaccinated $V\left( t \right)$, symptomatic infectious $I_{s}\left( t \right)$, asymptomatic infectious $I_{a}\left( t \right)$, and recovered $R\left( t \right)$. These classes are sets of nodes, which are disjoint within each component, such that at any given time t:

|  | $S^{V}\left( t \right)\cup I^{V}\left( t \right)\cup R^{V}\left( t \right)=N$ | (4a) |
| --- | --- | --- |
|  | $S_{V}\left( t \right)\cup S_{NV}\left( t \right)\cup V\left( t \right)\cup I_{s}\left( t \right)\cup I_{a}\left( t \right)\cup R\left( t \right)=N,$ | (4b) |

where $N$ is the set of the network nodes. We define state functions $c_{u}\left( t \right)$ that serve as an indicator that a user $u$ belongs to compartment C at time $t$*:*

|  | $c_{u}\left( t \right)=\left\{ \begin{aligned} 1 if u\in C at time t \\ 0 otherwise \end{aligned} \right.$. | (5) |
| --- | --- | --- |

Likewise, we define $i_{u}^{V}\left( t \right), r_{u}^{V}\left( t \right),s_{u}^{V}\left( t \right), s_{V,u}\left( t \right), {s_{NV,u}\left( t \right),i}_{a,u}\left( t \right),i_{s,u}(t),v_{u}(t), r_{u}(t)$.

## Model transitioning

### Vaccination component

We assumed that the adoption of the vaccination-uptake behavior could be the cause of either social influence, through its contacts in the network, or by non-social influence, which does not depend on the network. We used the Bass-SIR compartmental framework to model the vaccination behavior (6), where social adoption is considered as *internal influences*, and the non-social adoption is considered as *external influences*. Following the Bass model (7, 8), the probability of an individual $m$ in the $S^{V}$ compartment to become vaccinated at time interval $\left( t,t+\Delta t \right)$ is:

|  | $\mathrm{Prob}\left( \begin{aligned} m\text{ is vaccinated in} \\ \left( t,t+\Delta t \right) \end{aligned} \right)=\left( p^{\left( j \right)}+\beta_{v}^{\left( j \right)}\sum_{n\neq m} {a_{nm}\cdot i}_{n}^{V}\left( t \right) \right)\Delta t, \Delta t\to0,$ | (6) |
| --- | --- | --- |

where the parameter $p^{\left( j \right)}$ is the rate that an individual in $S^{V}$ gets vaccinated due to non-social influences, and $\beta_{v}^{\left( j \right)}$ is the rate due to social influences for age group $j$. We referred to two age groups in the vaccination component: 0-18 years and >18 years. Recall that $a_{nm}$ is the component of the adjacency matrix $A$, such that $a_{mn}=1$ if individual $m$ is connected to individual $n,$ and $a_{mn}=0$ otherwise. The system of equations describing the vaccination component is:

|  | $\mathrm{Pro}b_{\Delta t\to0}\left( \begin{aligned} u\in I^{V} \\ \text{in }\left( t+\Delta t \right) \end{aligned} \right)=i_{u}^{V}\left( t \right)\cdot\left( 1-\gamma_{v}\cdot\Delta t \right)+\mathrm{Prob}\left( \begin{aligned} m\text{ is vaccinated in} \\ \left( t,t+\Delta t \right) \end{aligned} \right),$ | (7a) |
| --- | --- | --- |
|  | $\mathrm{Pro}b_{\Delta t\to0}\left( \begin{aligned} u\in R^{V} \\ \text{in }\left( t+\Delta t \right) \end{aligned} \right)=r_{u}^{V}\left( t \right)+i_{u}^{V}\left( t \right)\cdot\gamma_{v}\cdot\Delta t,$ | (7b) |
|  | $\mathrm{Pro}b_{\Delta t\to0}\left( \begin{aligned} u\in S^{V} \\ \text{in }\left( t+\Delta t \right) \end{aligned} \right)=s_{u}^{V}\left( t \right)\cdot\left( 1-\mathrm{Prob}\left( \begin{aligned} m\text{ is vaccinated in} \\ \left( t,t+\Delta t \right) \end{aligned} \right) \right).$ | (7c) |

At the beginning of the season, individuals are *susceptible* to the idea of becoming vaccinated and therefore start in the susceptible class $S^{V}\left( 0 \right)$. To initiate the process, we randomly infect 0.001 of the nodes in the network and transfer them to the *infectious* class $I^{V}\left( 0 \right)$. At each time step $t$, infectious individuals can infect each of their susceptible contacts with the idea of becoming vaccinated with probability $\beta_{v}^{\left( j \right)}$. If infected, individuals are transferred to the *infectious* class $I^{V}\left( t \right)$ and can infect their contacts in the network until they recover at a rate $\gamma_{v}$. After this infectious period, individuals remain recovered and transferred to the recovered class $R^{V}\left( t \right)$. These individuals can no longer infect others with the idea of becoming vaccinated until the end of the season. To account for non-social spontaneous infection (9) with the idea to become vaccinated, at each time step, a randomly chosen susceptible node is infected and transferred to the *infectious* class $I^{V}\left( t \right)$.

### Disease component

At the beginning of the season, individuals start from the *susceptible* class $S\left( 0 \right)$. Individuals who are naturally immune due to cross-reactive antibodies gained from previous exposures (proportion of $\left( 1-\zeta^{\left( j \right)} \right)$ of each age group $j$) are not included in the susceptible class and transitioned to the *recovered* class $R\left( 0 \right)$ at the beginning of the season. The transition of susceptible individuals to the *vaccinated* class $V\left( t \right)$ is based on the vaccination component of the model and takes into account the vaccine efficacy (see 2.1.3 inter-component interaction). Individuals in the vaccinated class are protected from the disease and cannot get infected. When the immunity of the vaccine wanes, at rate $\omega$, these individuals are transferred back to the susceptible class, and cannot get vaccinated again (see 2.1.3). Susceptible individuals can get infected with influenza from their contacts, which are in one of the *infectious* class, based on the force of infection (see 2.2 force of infection). Infectious individuals can be either asymptomatic ($I_{a}$ class) with probability $f$ or symptomatic ($I_{s}$ class) with probability $\left( 1-f \right)$. These individuals get recovered at rate $\gamma_{i}$, and transferred to the recovered class $R\left( t \right)$, where they are no longer infectious and are immune to the disease for the rest of the season (model equations in 2.4). The probability of an individual $m$ to become infected, that is the force of infection at time interval $\Delta t$ is:

|  | $\mathrm{Pro}b_{\Delta t\to0}\left( \begin{aligned} m\text{ infected in} \\ \left( t,t+\Delta t \right) \end{aligned} \right)=\left( \Lambda\left( t \right)\sum_{n\neq m} a_{nm}\cdot\left( \rho^{\left( asymp \right)}\cdot i_{a,n}\left( t \right)+\rho^{\left( symp \right)}\cdot i_{s,n}(t) \right) \right)\Delta t,$ | (8) |
| --- | --- | --- |

where $\Lambda\left( t \right)$ is the seasonal rate, and $\rho^{\left( k \right)}$ is the transmissibility based on the type of infection $k$ (symptomatic/asymptomatic). The system of equations describing the disease transmission component is:

|  | $\mathrm{Pro}b_{\Delta t\to0}\left( \begin{aligned} m\in I_{a} \\ \text{in }\left( t+\Delta t \right) \end{aligned} \right)=i_{a,m}\left( t \right)\cdot\left( 1-\gamma_{i}^{\left( asym,j \right)}\cdot\Delta t \right)+\left( s_{V,m}\left( t \right)+s_{NV,m}\left( t \right) \right)\cdot f\cdot\mathrm{Prob}\left( \begin{aligned} m\text{ infected in} \\ \left( t,t+\Delta t \right) \end{aligned} \right),$ | (9a) |
| --- | --- | --- |
|  | $\mathrm{Pro}b_{\Delta t\to0}\left( \begin{aligned} m\in I_{s} \\ \text{in }\left( t+\Delta t \right) \end{aligned} \right)=i_{s,m}\left( t \right)\cdot\left( 1-\gamma_{i}^{\left( symp,j \right)}\cdot\Delta t \right)+\left( s_{V,m}\left( t \right)+s_{NV,m}\left( t \right) \right)\cdot\left( 1-f \right)\cdot\mathrm{Prob}\left( \begin{aligned} m\text{ infected in} \\ \left( t,t+\Delta t \right) \end{aligned} \right),$ | (9b) |
|  | $\mathrm{Pro}b_{\Delta t\to0}\left( \begin{aligned} m\in V \\ \text{in }\left( t+\Delta t \right) \end{aligned} \right)=\left( v_{m}\left( t \right)\cdot\left( 1-\omega\cdot\Delta t \right)+s_{NV,m}\left( t \right)\cdot\left( \psi\cdot\eta\right)\cdot\left( i_{m}^{V}\left( t \right)+ r_{m}^{V}\left( t \right) \right)\cdot\Delta t \right),$ | (9c) |
|  | $\mathrm{Pro}b_{\Delta t\to0}\left( \begin{aligned} m\in S_{V} \\ \text{in }\left( t+\Delta t \right) \end{aligned} \right)=s_{V,m}\left( t \right)+v_{m}\left( t \right)\cdot\left( \omega\cdot\Delta t \right)-s_{V,m}\left( t \right)\cdot Prob\left( \begin{aligned} m\text{ infected in} \\ \left( t,t+\Delta t \right) \end{aligned} \right),$ | (9d) |
|  | $\mathrm{Pro}b_{\Delta t\to0}\left( \begin{aligned} m\in S_{NV} \\ \text{in }\left( t+\Delta t \right) \end{aligned} \right)=s_{NV,m}\left( t \right)\cdot\left( 1-\left( \psi\cdot\eta\right)\cdot\left( i_{m}^{V}\left( t \right)+ r_{m}^{V}\left( t \right) \right)\cdot\Delta t \right)-s_{NV,m}\left( t \right)\cdot\mathrm{Prob}\left( \begin{aligned} m\text{ infected in} \\ \left( t,t+\Delta t \right) \end{aligned} \right),$ | (9e) |
|  | $\mathrm{Pro}b_{\Delta t\to0}\left( \begin{aligned} m\in R \\ \text{in }\left( t+\Delta t \right) \end{aligned} \right)=r_{m}\left( t \right)+\left( i_{a,m}\left( t \right)\cdot\gamma_{i}^{\left( asym,j \right)}+i_{s,m}\left( t \right)\cdot\gamma_{i}^{\left( symp,j \right)} \right)\cdot\Delta t.$ | (9f) |

### Inter-component interaction

The vaccination component determines the transition of individuals from the susceptible class of the disease component 𝑆 to the vaccinated class of the disease component 𝑉. Specifically, when individuals are infected with the idea of becoming vaccinated, i.e., when transferred to the infectious class of the vaccination component $I^{V}$, they get vaccinated. If the vaccine is effective (with probability $\eta$), and after the antibodies are developed (10) at rate $\psi$, the individual is protected from the disease. The individual is transferred to the *vaccinated* class of the disease component $V\left( t \right)$ if at that time step $t$ he belongs to the *non-vaccinated* *susceptible* class of the disease component $S_{NV}\left( t \right)$. Otherwise, if the individual is already infected or recovered (either after an infection or due to cross-reactivity), he stays in the same class as the vaccination is not relevant. Vaccinated individuals who became susceptible due to the vaccine-induced immunity waning are transitioned to $S_{V}\left( t \right)$. Individuals in $S_{V}\left( t \right)$ will not get vaccinated again, as they are already in the *recovered* class of the vaccination component, and therefore cannot get infected again with the idea of getting vaccinated.

## The Seasonal rate

The probability at which an individual acquires influenza infection given a contact depends on seasonality and the infectiousness of the infected contact. The peak incidence of influenza typically strikes during the winter. Thus, we included a general seasonal variation in the force of infection of the model. Given a contact with an infected host, the logarithm of the infectious viral load is correlated with the transmissibility of several respiratory viruses (11). The logarithm of the viral load, or the transmissibility of an individual, depends on the infection type, namely, symptomatic or asymptomatic. Taken together, the rate at which an individual acquires influenza infection given a contact is given by:

|  | $\Lambda\left( t \right)=\beta_{i}\cdot\left( 1+cos\left( \frac{2\pi t}{365}+\phi\right) \right),$ | (10) |
| --- | --- | --- |

where $\beta_{i}$ is the infection susceptibility rate, and $\phi$ is the seasonality offset (Table S2).

## Model parameters

### Fixed parameters

The model's fixed parameters are described in Table S2. The values of the parameters are based on relevant literature. All the fixed parameters are relevant to the disease component.

| **Table S2.** Fixed parameters used in the disease component of the model. All probabilities are daily, $j$ indicates the age group (0-18, >18) and k indicates the infection type (symptomatic/asymptomatic) | | | |
| --- | --- | --- | --- |
| **Parameter** | **Description** | **Value** | **Justification** |
| $\gamma_{i}^{\left( k,j \right)}$ | Recovery rate | $\gamma_{i}^{\left( asym,j \right)}=\frac{1}{3.2}$  $\gamma_{i}^{\left( symp,0-18 \right)}=\frac{1}{0.675}$  $\gamma_{i}^{\left( symp,0-18 \right)}=\frac{1}{4.5}$ | (12, 13) |
| $\psi$ | Probability of vaccine-induced immunity to become effective | $\frac{1}{14}$ | (10) |
| $\omega$ | Probability of vaccine-induced immunity waning | $\frac{1}{111}$ | (14) |
| $\eta$ | Influenza vaccine efficacy | $0.45$ | (15) |
| $\rho^{\left( k \right)}$ | Transmissibility (logarithm of the viral load) | $\rho^{\left( asymp \right)}=4.5$  $\rho^{\left( symp \right)}=5.5$ | (11, 16) |
| $\zeta^{\left( j \right)}$ | Probability to be protected against vaccination due to exposure in previous seasons (due to cross-reactivity) | $\zeta^{\left( 0-18 \right)}=0.2$  $\zeta^{\left( 18 \right)}=0.7$ | (17–20) |
| $f$ | Probability of asymptomatic cases | 0.191 | (21–23) |

### Calibrated parameters

The model requires three free parameters for the vaccination component and two free parameters per season for the disease component (Table S3).

| **Table S3.** Calibrated parameters. All probabilities are daily. | | |
| --- | --- | --- |
| **Parameter** | **Description** | **Comments** |
| **Vaccination component** | | |
| $\beta_{v}^{\left( 0-18 \right)}$ | vaccination susceptibility rate (given a contact) |  |
| $\beta_{v}^{\left( 18 \right)}$ |  |  |
| $\gamma_{v}$ | recovery probability |  |
| **Disease component (per season)** | | |
| $\beta_{i}$ | Infection susceptibility rate (given a contact) |  |
| $\phi$ | Seasonality offset | Estimated between the 25^th^ and 35^th^ weeks of the season |

## Model calibration

### The data

To estimate the unknown parameters, we calibrated the model to weekly vaccination uptake data as well as influenza and influenza-like-illness (ILI) diagnoses by age and geographic location. The data was collected by the Maccabi Health Maintenance Organization (HMO), the second largest HMO in Israel. These electronic medical records (EMR) include longitudinal data of 250,000 members (randomly assigned), between 2007 and 2017. Each patient is affiliated with one of 138 clinics of Maccabi. Patients are affiliated with their home subdistrict based on the location of their clinic. Demographic data is available as well, including age.

*Vaccination uptake data*

The EMRs specifies for each individual her influenza vaccination uptake, including the day of vaccination in each season, if any. These data were aggregated by subdistrict and age group and by week of the season to receive the weekly proportion of newly vaccinated for each subdistrict and age group (the red dots display these data in Main text, Fig. 2A-D, Fig. S5, and Fig. S6). For the calibration of the vaccination component parameters, we averaged these data over the seven seasons to receive the average weekly proportion of newly vaccinated for each subdistrict and age group.

*Influenza and ILI diagnosis data*

The EMRs also include respiratory infection diagnoses, including influenza and influenza-like-illness (ILI). For each patient, the data include the dates of the diagnoses, if any, and the diagnosed disease. The types of respiratory infection diagnoses that were used for this purpose are detailed in Table S4. These data were aggregated by subdistrict and age group and by week of the season to receive the weekly proportion of newly infected for each subdistrict and age group. To account for unreported cases, the influenza weekly diagnoses data was adjusted to fit actual attack rates by age group reported in previous studies in Israel (24). The blue dots display these data in the Main text, Fig. 2A-D, Fig. S5, and Fig. S6.

| **Table S4.** Respiratory infection diagnoses by the International Classification of Diseases codes (ICD-9) | |
| --- | --- |
| **ICD-9 code** | **Description** |
| 487 | influenza |
| 487.0 | Influenza with pneumonia |
| 487.1 | Influenza with other respiratory manifestations |
| 478.9 | Other and unspecified diseases of the upper respiratory tract |
| 482.2 | 1. Pneumonia due to Haemophilus influenzae [H. influenzae] |

### Likelihood function

Assuming Poisson distribution, the likelihood function of the model is given by:

|  | $L\left( \theta\right)=\prod_{c,s,j,w} \frac{e^{-\hat{\lambda}_{csjw}}\cdot\left( \hat{\lambda}_{csjw} \right)^{d_{csjw}}}{d_{csjw}!},$ | (13) |
| --- | --- | --- |

where $\theta$ is the set of parameters, $d_{csjw}$ represents the weekly data of component $c$ (i.e., the weekly vaccination uptake/influenza and ILI diagnoses), for subdistrict $s$, age group $j$ and week $w$), and $\hat{\lambda}_{csjw}$ is the Poisson distribution parameter estimated from the model for component $c$ (vaccination/disease) for each subdistrict $s$, age group $j,$ and week $w$. The set of parameters is $\theta=\left( \beta_{v}^{\left( 0-18 \right)},\beta_{v}^{\left( 18 \right)},\gamma_{v},\beta_{i},\phi\right)$.

Maximizing the likelihood is equivalent to maximizing the log-likelihood. Therefore, due to computational issues, we maximized the expression:

|  | $\max_{\theta}L\left( \theta\right)\leftrightarrow\max_{\theta}ln\left( L\left( \theta\right) \right)\leftrightarrow\max_{\theta}\sum_{c,s,j,w} \left( -\hat{\lambda}_{csjw}+d_{csjw}\cdot ln\left( \hat{\lambda}_{csjw} \right) \right).$ | (14) |
| --- | --- | --- |

The Poisson distribution daily rate parameter $\hat{\lambda}_{csjt}$ is estimated using the model according to the pseudocode described in Algorithm S1 and Algorithm S2 for the vaccination component and the disease component, respectively. We aggregate the daily rate parameter weekly to receive the estimation of the weekly Poisson rate parameter $\hat{\lambda}_{csjw}$.

| **Algorithm S1.** estimation of the Poisson distribution rate parameter for the vaccination component |
| --- |
| **Initialize** $\lambda_{vsjw}=0 \forall s,j,t$  **for** $t\in T_{v}$:  **for** $n$ $\in I^{V}\left( t-1 \right)$:  **for** $c$ $\in\left( C\left( n \right)\cap S^{V}\left( t \right) \right)\setminus I_{new}^{V}\left( t \right)$:  $\lambda_{v,s_{c},j_{c},w}\leftarrow\lambda_{v,s_{c},j_{c},w}+\beta_{v}^{\left( j_{c} \right)}$  **end for**  **end for**  **end for** |
| ***** $T_{v}$ is the set of time steps relevant for the vaccination season, $C\left( n \right)=\left\{ c\vee\left( c,n \right)\in E \right\}$ is the set of contacts of node $n$, $j_{c}$ is the subdistrict of the contact node $c$, $j_{c}$ is the age group of the contact node $c$ |

| **Algorithm S2.** estimation of the Poisson distribution rate parameter for the disease component |
| --- |
| **Initialize** $\lambda_{isjw}=0\forall s,j,t$  **for** $t\in T_{i}$:  **for** $n$ $\in I_{s}\left( t-1 \right)\cup I_{a}\left( t-1 \right)$:  **for** $c$ $\in\left( C\left( n \right)\cap S\left( t \right) \right)\setminus\left( I_{s,new}\left( t \right)\cup I_{a,new}\left( t \right) \right)$:  $\lambda_{i,s_{c},j_{c},w}\leftarrow\lambda_{i,s_{c},j_{c},w}+\Lambda\left( t \right)$  **end for**  **end for**  **end for** |
| ***** $T_{i}$ is the set of time steps relevant for the influenza season, $C\left( n \right)=\left\{ c\vee\left( c,n \right)\in E \right\}$ is the set of contacts of node $n$, $j_{c}$ is the subdistrict of the contact node $c$, $j_{c}$ is the age group of the contact node $c$ |

### The calibration process

The model parameters were calibrated in two stages. First, we calibrated the vaccination component parameters, independently of the disease component. We chose the parameters that maximize the log-likelihood described in [11] against the data of the average weekly proportion of newly vaccinated by subdistrict and age described in 2.5.1. The Poisson rate parameter was calculated based on the model, as described in Algorithm S1. We use these parameters for all seven seasons.

At the second stage, we calibrate the disease component parameters for each season separately, using the calibrated parameters of the vaccination component. We chose the parameters that maximize the log-likelihood described in [11] against the data of the average weekly proportion of newly infected by subdistrict and age described in 2.5.1. The Poisson rate parameter was calculated based on the model, as described in Algorithm S2.

Due to the variance resulted from the network, we used a few realizations for each set of parameters. The average log-likelihood value was chosen to represent this set of parameters for the maximization.

### Maximum likelihood parameter estimates

| **Table S5.** Maximum likelihood parameter estimates for the vaccination and the disease components of the network-based model | | | |
| --- | --- | --- | --- |
| **parameter** | | **description** | **value** |
| **Vaccination component** | | | |
| $\beta_{v}^{\left( 0-18 \right)}$ | | infection probability (given a contact) | 0.0063 |
| $\beta_{v}^{\left( 18 \right)}$ | |  | 0.0165 |
| $\gamma_{v}$ | | recovery probability | 0.2084 |
| **Disease component (per season)** | | | |
| $\beta_{i}$ | 2011 | Infection parameter (given a contact) | 0.00174 |
|  | 2012 |  | 0.00151 |
|  | 2013 |  | 0.00158 |
|  | 2014 |  | 0.00155 |
|  | 2015 |  | 0.00156 |
|  | 2016 |  | 0.00166 |
|  | 2017 |  | 0.00151 |
| $\phi$ | 2011 | Seasonality offset | -3.850 |
|  | 2012 |  | -3.862 |
|  | 2013 |  | -3.811 |
|  | 2014 |  | -4.148 |
|  | 2015 |  | -3.936 |
|  | 2016 |  | -3.842 |
|  | 2017 |  | -3.506 |

### Detailed fit

The model results of the weekly vaccinated individuals, and newly infected individuals are displayed against the relevant data (see 2.5.1.) in the Main text Fig. 2A-D, Fig. S5, and Fig. S6. To describe the variance in the results resulted from the network, we display the results of 200 realizations of the model. The bold line in each season represents the realization with the median log-likelihood value out of the 200 realizations.

| 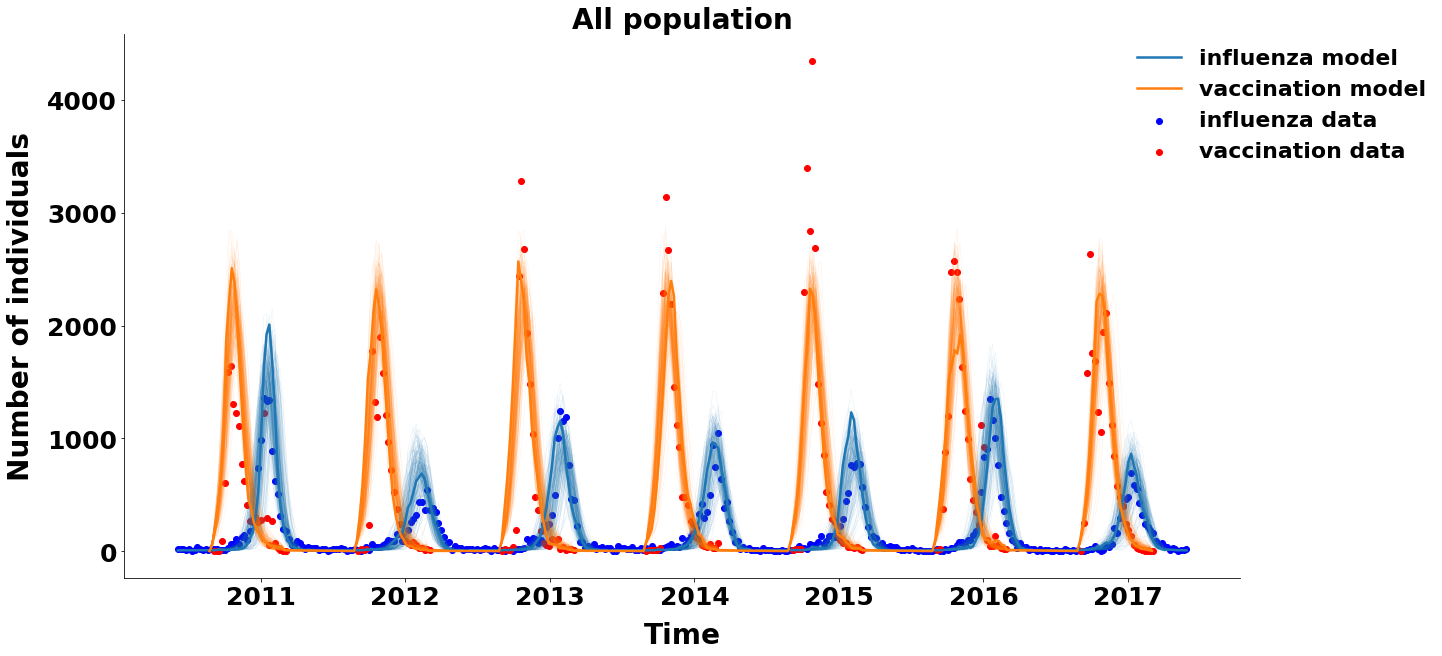 |
| --- |
| **Fig. S5.** Overall model fit. Time series of weekly influenza vaccination uptake, weekly influenza, and ILI diagnoses and model fit of both vaccination and disease components for 2011-2017, aggregated for the whole population. |

| 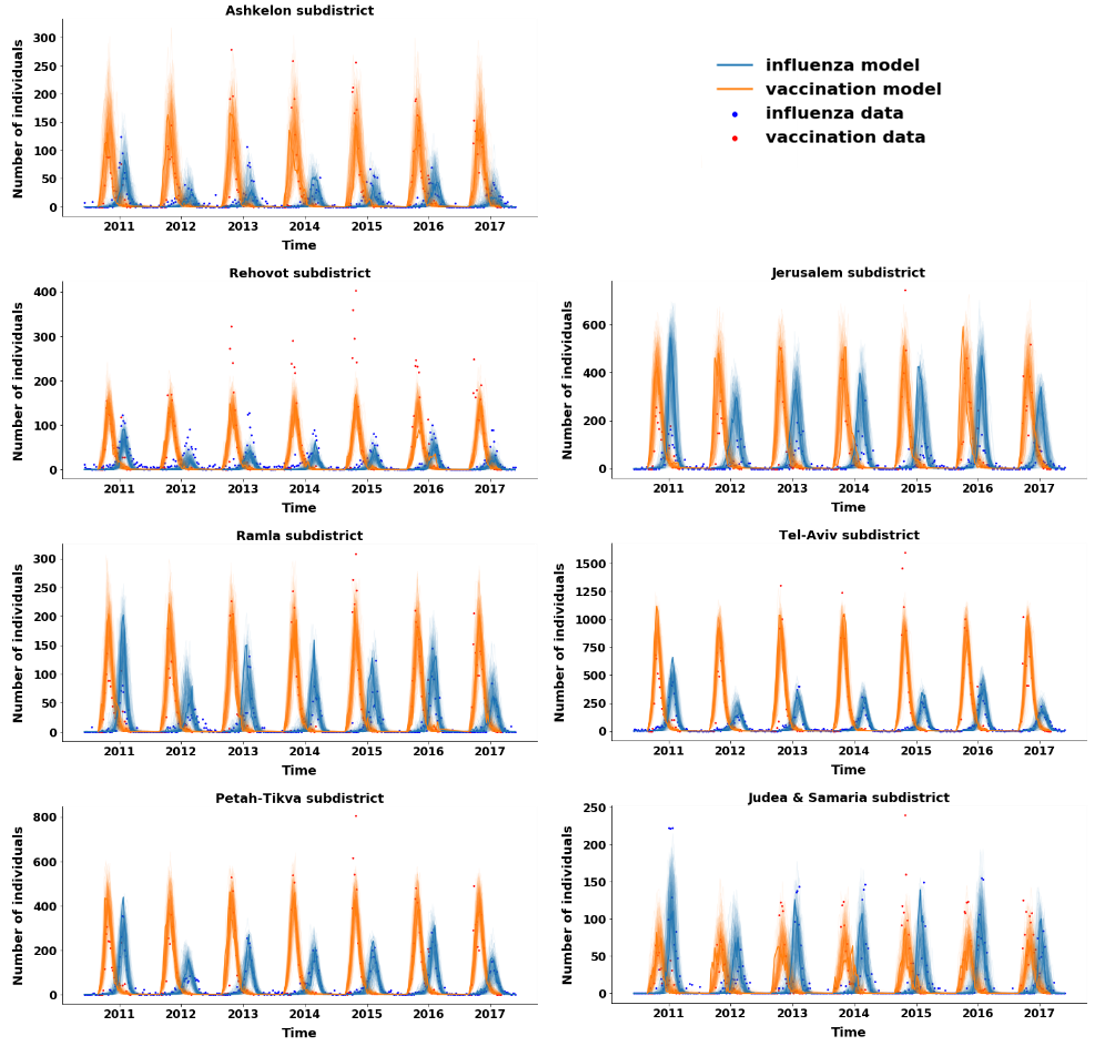 |
| --- |
| **Fig. S6.** Model fit by subdistrict. Time series of weekly influenza vaccination uptake, weekly influenza, ILI diagnoses, and model fit of both vaccination and disease components for 2011-2017, aggregated for each of the seven subdistricts. |

### Correlation fit

We used Pearson correlation to compare the model's weekly number of vaccinated and infected individuals in each subdistrict and age group with vaccination uptake, influenza, and ILI diagnoses data, respectively. For each season, we calculated the average of the correlation values weighted by the population size of each subdistrict and age group. Finally, we averaged the results of all seven seasons to receive the final correlation fit value.

## Comparison with a homogenous model

We compare our network-based model to a simpler homogenous model. This model ignores the network structure and assumes mass action mixing (5), i.e., individuals contact each of the other individuals at the same probability, and the force of infection is proportional to the number of infectious individuals at time $t$. To compare between the models, the homogenous model is based on the same network with all nodes connected to each other. The probability of contact between nodes differs by age, according to the contact probability matrix aggregated by age group. The homogenous model uses the same number of parameters for both the vaccination and the disease components. The parameters were recalibrated for the homogenous model, as described in 2.5.3. For this comparison, we calibrated the disease component parameters for one season (2016) and the vaccination component parameters (Table S6).

| **Table S6.** Maximum likelihood parameter estimates for the vaccination and the disease components of the homogenous model | | | |
| --- | --- | --- | --- |
| **parameter** | | **description** | **value** |
| **Vaccination component** | | | |
| $\beta_{v}^{\left( 0-18 \right)}$ | | infection probability (given a contact) | 0.0000045 |
| $\beta_{v}^{\left( 18 \right)}$ | |  | 0.0000086 |
| $\gamma_{v}$ | | recovery probability | 0.39 |
| **Disease component (per season)** | | | |
| $\beta_{i}$ | 2016 | Infection parameter | 0.00000085 |
| $\phi$ | 2016 | Seasonality offset | -4.017 |

We compared the network-based model to the homogenous model using the Akaike information criterion (AIC) derived from information theory. The network-based model yields a higher AIC value compared to the homogenous model.

# Optimization of influenza vaccination program

## Base case

The base case for the optimization process is the case of the state of Israel, with average vaccination uptake of ~20%. The calibrated parameters (detailed in Table S5) describes the base case, as the parameters were calibrated to data of the population of Israel (see 2.5.1). In the case displayed in Main text, Fig. 2A-D, Fig. S5, and Fig. S6, the vaccination program start date is at the beginning of September.

In order to optimize vaccination program timing, we examined different start dates for the vaccination program (from July 1 to December 1 in half-month steps). We used the coupled transmission model to simulate the vaccination and disease propagation over the seven seasons for each of the program start dates. Specifically, we started initial infectious individuals $I^{V}\left( 0 \right)$, as well as the random infection with the idea of becoming vaccinated at a different time in the season. We simulate the seven seasons 100 times and evaluate the average yearly attack rate over all the simulations and the seven seasons. Then, we compare the attack rates of the different model settings to find the optimal vaccination program start date, namely the start date corresponding to the lowest average yearly attack rate. The results are displayed in the bold blue line in Main text Fig. 4.

## Sensitivity analysis

### Vaccination coverage

We performed a sensitivity analysis of the overall vaccination coverage by optimizing the vaccination program start date (described in 3.1) for four different values of overall vaccination coverage (10, 20, 30, and 45%), and comparing the optimal start dates (Main text, Fig 4A). Different values of overall vaccination coverage were obtained by modifying the infection probability parameters of the vaccination component. Parameters values were increased or decreased (both infection probability parameters by the same proportion) to receive the wanted overall vaccination coverage. The modified values of the infection probability parameters of the vaccination component are described in Table S7. The results of the sensitivity analysis are displayed in Main text in Fig. 4A.

| **Table S7.** Modified values of vaccination component infection probability parameters by wanted overall vaccination coverage. | | |
| --- | --- | --- |
| **Overall vaccination coverage** | $\beta_{v}^{\left( 0-18 \right)}$ | $\beta_{v}^{\left( 18 \right)}$ |
| **Base case (~20%)** | 0.0063 | 0.0165 |
| **~10%** | 0.0054 | 0.01402 |
| **~30%** | 0.0078 | 0.0203 |
| **~45%** | 0.0107 | 0.028 |

### Vaccine immunity waning time

We performed a sensitivity analysis of the vaccine-induced immunity waning time by optimizing the vaccination program start date (described in 3.1) for four different values of waning time (60, 111, 150, and 240 days), and comparing the optimal start dates (Main text, Fig 4B). Different values of waning time were obtained by modifying the probability of vaccine-induced immunity waning parameter $\omega$ (Table S2, fixed parameters). The modified values of this parameter are described in Table S8. The results of the sensitivity analysis are displayed in Main text in Fig. 4B.

| **Table S8.** Modified values of vaccine waning time. | | |
| --- | --- | --- |
| **Vaccine immunity waning time** | $\omega$ |  |
| **Base case (111 days)** | $\frac{1}{111}$ |  |
| **60 days** | $\frac{1}{60}$ |  |
| **150 days** | $\frac{1}{150}$ |  |
| **240 days** | $\frac{1}{240}$ |  |

# Network model pseudocode and complexity

The classes of the vaccination component and of the disease component are represented as disjoint sets containing the relevant nodes. The network is represented a directed graph $G=\left( N,E \right)$.

## Vaccination component

The vaccination component is composed of the following equation system:

| $S_{v}\left( t \right)=S_{v}\left( t-1 \right)\setminus I_{v}^{new}\left( t \right)$  $I_{v}\left( t \right)=I_{v}\left( t-1 \right)\cup I_{v}^{new}\left( t \right)\setminus R_{v}^{new}\left( t \right)$  $R_{v}\left( t \right)=R_{v}\left( t-1 \right)\cup R_{v}^{new}\left( t \right)$  Initial conditions:  $S_{v}\left( 0 \right)=N\setminus I_{v}\left( 0 \right)$  $I_{v}\left( 0 \right)=\left\{ \left( 0.001\cdot\left\vert N \right\vert\right) random nodes \right\}$  $R_{v}\left( 0 \right)=\left\{ \right\}$ |  |
| --- | --- |

Where $I_{v}^{new}\left( t \right)$ and $R_{v}^{new}\left( t \right)$ are calculated according to the pseudocode described in Algorithm S3 and Algorithm S4, respectively.

| **Algorithm S3.** Calculation of *newly infected* in the vaccination component at time step t $\left( I_{v}^{new}\left( t \right) \right)$ |
| --- |
| **Initialize** $I_{v}^{new}\left( t \right)=$ { }  **for** $n$ $\in$ $I_{v}\left( t-1 \right)$:  **for** $c$ $\in$ $\left( C\left( n \right)\cap S_{v}\left( t \right) \right)\setminus I_{v}^{new}\left( t \right)$:  **with probability** $\beta_{v}^{\left( j_{c} \right)}$:  **add** $c$ to $I_{v}^{new}\left( t \right)$  **end for**  **end for**  r $\leftarrow$ **sample** a random node from $S_{v}\left( t \right)\setminus I_{v}^{new}\left( t \right)$  **add** r **to** $I_{s}^{new}\left( t \right)$ |
| *****$C\left( n \right)=\left\{ c \vert\left( c,n \right)\in E \right\}$ is the set of contacts of node $n$, $j_{c}$ is the age group of the contact node $c$ |

| **Algorithm S4.** Calculation of *newly recovered* in the vaccination component at time step t $\left( R_{v}^{new}\left( t \right) \right)$ |
| --- |
| **Initialize** $R_{v}^{new}\left( t \right)=$ { }  **for** $n$ $\in I_{v}\left( t-1 \right)$:  **with probability** $\gamma_{v}$:  **add** $n$ to $R_{v}^{new}\left( t \right)$  **end for** |

## Disease component

The vaccination component is composed of the following equation system:

| $S_{i}\left( t \right)=S_{i}\left( t-1 \right)\cup S_{i}^{new}\left( t \right)\setminus\left( I_{s}^{new}\left( t \right)\cup I_{a}^{new}\left( t \right)\cup V^{new}\left( t \right) \right)$  $V\left( t \right)=V\left( t-1 \right)\cup V^{new}\left( t \right)\setminus S_{i}^{new}\left( t \right)$  $I_{s}\left( t \right)=I_{s}\left( t-1 \right)\cup I_{s}^{new}\left( t \right)\setminus R_{i}^{new}\left( t \right)$  $I_{a}\left( t \right)=I_{a}\left( t-1 \right)\cup I_{a}^{new}\left( t \right)\setminus R_{i}^{new}\left( t \right)$  $R_{i}\left( t \right)=R_{i}\left( t-1 \right)\cup R_{i}^{new}\left( t \right)$  Initial conditions:  $S_{i}\left( 0 \right)=N\setminus R_{i}\left( 0 \right)$ ; $\left\vert S_{i}\left( 0 \right) \right\vert=\left( 1-\zeta^{\left( j \right)}-\zeta^{\left( j \right)} \right)\cdot\left\vert N \right\vert$  $V\left( 0 \right)=\{ \}$  $I_{s}\left( 0 \right)=\left\{ \right\}$  $I_{a}\left( 0 \right)=\{ \}$  $R_{i}\left( 0 \right)=\{immunded due to cross reactivity\}$ ; $\left\vert R_{i}\left( 0 \right) \right\vert=\left( \zeta^{\left( j \right)}+\zeta^{\left( j \right)} \right)\cdot\left\vert N \right\vert$ |  |
| --- | --- |

Where $S_{i}^{new}\left( t \right)$, $I_{s}^{new}\left( t \right), I_{a}^{new}\left( t \right)$, $R_{i}^{new}\left( t \right)$ and $V^{new}\left( t \right)$are calculated according to the pseudocode described in Algorithms S5-S8 respectively.

| **Algorithm S5.** Calculation of *newly susceptible* in the disease component at time step t $\left( S_{i}^{new}\left( t \right) \right)$ |
| --- |
| **Initialize** $S_{i}^{new}\left( t \right)=$ { }  **for** $n$ **in** $V\left( t-1 \right)$:  **with probability** $\omega$:  **add** $n$ to $S^{new}\left( t \right)$  **end for** |

| **Algorithm S6.** Calculation of *newly infected* in the disease component at time step t ($I_{s}^{new}\left( t \right)$ and $I_{a}^{new}\left( t \right)$) |
| --- |
| **Initialize** $I_{s}^{new}\left( t \right)=$ { }, $I_{a}^{new}\left( t \right)=$ { }  **for** $n$ $\in$ $I_{s}\left( t-1 \right)\cup I_{a}\left( t-1 \right)$:  **for** $c$ $\in\left( C\left( n \right)\cap S_{i}\left( t \right) \right)\setminus\left( I_{s}^{new}\left( t \right)\cup I_{a}^{new}\left( t \right) \right)$:  **with probability**$\Lambda\left( t \right)$:  **with probability** $f$:  **add** $c$ **to** $I_{a}^{new}\left( t \right)$  **otherwise**:  **add** $c$ **to** $I_{s}^{new}\left( t \right)$  **end for**  **end for**  r $\leftarrow$ **sample** a random node from $S_{i}\left( t \right)\setminus\left( I_{s}^{new}\left( t \right)\cup I_{a}^{new}\left( t \right) \right)$  **add** r **to** $I_{s}^{new}\left( t \right)$ |
| *****$C\left( n \right)=\left\{ c \vert\left( c,n \right)\in E \right\}$ is the set of contacts of node $n$ |
| **Algorithm S7.** Calculation of *newly recovered* in the disease component at time step t $\left( R_{i}^{new}\left( t \right) \right)$ |
| **Initialize** $R_{i}^{new}\left( t \right)=$ { }  **for** $n$ $\in I_{s}\left( t-1 \right)\cup I_{a}\left( t-1 \right)$:  **with probability** $\gamma_{i}^{\left( k,j_{n} \right)}$:  **add** $n$ **to** $R_{i}^{new}\left( t \right)$  **end for** |
| *****$j_{n}$ is the age group of the node $n$ |

| **Algorithm S8.** Calculation of *newly vaccinated* in the disease component at time step t $\left( V^{new}\left( t \right) \right)$ |
| --- |
| **Initialize** $V^{new}\left( t \right) =$ { }  **for** $n$ $\in I_{v}\left( t-1 \right)\cup R_{v}\left( t-1 \right)\setminus\left( V_{e}\cup V_{ne}\cup R_{i}\left( t-1 \right) \right)$:  **with probability** $\psi$:  **with probability** $\eta$**:**  **add** $n$ to $V^{new}\left( t \right)$  **add** $n$ to $V_{e}$  **otherwise:**  **add** $n$ to $V_{ne}$  **end for** |
| *****$V_{e}$ and $V_{ne}$ are dummy sets. $V_{e}$ include nodes with an effective vaccination and $V_{ne}$ include nodes with a non-effective vaccination (due to the vaccine efficacy) |

## Computational complexity

Here we present a complexity analysis of the vaccination component of the model. Each compartment of the model ($S^{V}, I^{V}, R^{V})$ is represented by a set of nodes (the initial values of each set is described in 4.2). At each step, two transitions need to be done, from $S^{V}$ to $I^{V}$ and from $I^{V}$ to $R^{V}$. For these transitions, the calculation of newly infected at time t and newly recovered in time t is required. The calculation of newly infected individuals is described in algorithm S3 and includes going over all the infected nodes at time t ($I^{V}\left( t \right)$), and for each of them going over their susceptible contacts in the network $\left( C\left( n \right)\cap S_{v}\left( t \right) \right)$. Nodes are infected with probability $\beta$ and added to a dedicated set of newly infected. The $S^{V}$ and $I^{V}$ will be updated accordingly. In addition, few random nodes are added to the newly infected set to account for internal influences. The complexity of this stage can be described by:

$$T\cdot[O\left( N\cdot C \right)+2\cdot O\left( N \right)]=O(T\cdot C\cdot N)$$

Where $T$ is the number of time steps, $N$ is the number of nodes in the network, and $C$ is the number of contacts of a node (for the complexity analysis, we can take the average or the maximal value in the network). The first expression corresponds to the double loop, the maximal size of $I^{V}$ is the size of the network (note that in this case there won’t be any susceptible contacts). The second expression corresponds to the update of the set.

The calculation of the newly recovered is done by going over the infected individuals and adding nodes to a dedicated set of newly recovered with probability $\gamma$. The $R^{V}$ and $I^{V}$ will be updated accordingly. The complexity of this stage can be described by:

$$T\cdot[O\left( N \right)+2\cdot O\left( N \right)]=O\left( T\cdot N \right)$$

The first expression corresponds to the loop and the second expression corresponds to the set update.

In this study, we used a contact network with $N=100,000$ nodes. The average number of contacts in the network is 15 (16.83 for the 0-18 age group, and 12.14 for the >18 age group). We simulated 7 years between 2011-2017 with time steps of days (i.e., $T=7\cdot365=2555)$.

Moreover, another issue that affects the computational complexity of the simulation is the stochasticity of the network-based model, which requires running few iterations and calculating the mean and standard deviation (as opposed to the determinism of a homogenous differential equations model).

Running one iteration of the coupled model takes on average 25.4 seconds $\pm$ 300 ms (using Intel Core i9-7920X CPU).

**References**

1. D. Naboulsi, M. Fiore, S. Ribot, R. Stanica, Large-scale Mobile Traffic Analysis: a Survey. *Surv. Commun. Surv. Tutorials* **18**, 124–161 (2016).

2. S. Brin, L. Page, The PageRank Citation Ranking: Bringing Order to the Web. *BMC Syst. Biol.* **4 Suppl 2**, S13 (2010).

3. A. N. Langville, C. D. Meyer, A Survey of Eigenvector Methods for Web Information Retrieval. *SIAM Rev.* **47**, 135–161 (2005).

4. K. Prem, A. R. Cook, M. Jit, Projecting social contact matrices in 152 countries using contact surveys and demographic data. *PLoS Comput. Biol.* **13**, e1005697 (2017).

5. E. Vynnycky, R. White, *An Introduction to Infectious Disease Modelling* (Oxford University Press, USA, 2010).

6. G. Fibich, Bass-SIR model for diffusion of new products in social networks. *Phys. Rev. E* **94**, 032305 (2016).

7. F. M. Bass, A New Product Growth for Model Consumer Durables. *Manage. Sci.* **15**, 215–227 (1969).

8. G. Fibich, R. Gibori, Aggregate Diffusion Dynamics in Agent-Based Models with a Spatial Structure. *Oper. Res.* **58**, 1450–1468 (2010).

9. A. L. Hill, D. G. Rand, M. A. Nowak, N. A. Christakis, Infectious disease modeling of social contagion in networks. *PLoS Comput. Biol.* **6**, 1000968 (2010).

10. CDC, Misconceptions about Seasonal Flu and Flu Vaccines (2018).

11. R. Tellier, Aerosol transmission of influenza A virus: A review of new studies. *J. R. Soc. Interface* **6** (2009).

12. S. Ng, *et al.*, The Timeline of Influenza Virus Shedding in Children and Adults in a Household Transmission Study of Influenza in Managua, Nicaragua. *Pediatr. Infect. Dis. J.* **35**, 583–586 (2016).

13. J. E. Fielding, H. A. Kelly, G. N. Mercer, K. Glass, Systematic review of influenza A(H1N1)pdm09 virus shedding: duration is affected by severity, but not age. *Influenza Other Respi. Viruses* **8**, 142–150 (2014).

14. E. Kissling, *et al.*, I-MOVE multicentre case–control study 2010/11 to 2014/15: Is there within-season waning of influenza type/subtype vaccine effectiveness with increasing time since vaccination? *Eurosurveillance* **21**, 30201 (2016).

15. CDC, Seasonal Influenza Vaccine Effectiveness, 2004-2018 (2018).

16. D. K. M. Ip, *et al.*, Viral Shedding and Transmission Potential of Asymptomatic and Paucisymptomatic Influenza Virus Infections in the Community. *Clin. Infect. Dis.* **64**, 736–742 (2017).

17. B. B., *et al.*, Age-related prevalence of cross-reactive antibodies against influenza a(H3N2) variant virus, Germany, 2003 to 2010. *Eurosurveillance* **20**, 9 (2015).

18. K. Hancock, *et al.*, Cross-Reactive Antibody Responses to the 2009 Pandemic H1N1 Influenza Virus. *N. Engl. J. Med.* **361**, 1945–1952 (2009).

19. , Antibodies Cross-Reactive to Influenza A (H3N2) Variant Virus and Impact of 2010–11 Seasonal Influenza Vaccine on Cross-Reactive Antibodies — United States (January 29, 2020).

20. M. Mandelboim, *et al.*, Significant cross reactive antibodies to influenza virus in adults and children during a period of marked antigenic drift. *BMC Infect. Dis.* **14** (2014).

21. L. Furuya-Kanamori, *et al.*, Heterogeneous and dynamic prevalence of asymptomatic influenza virus infections. *Emerg. Infect. Dis.* **22**, 1052–1056 (2016).

22. N. H. L. Leung, C. Xu, D. K. M. Ip, B. J. Cowling, The fraction of influenza virus infections that are asymptomatic: A systematic review and meta-analysis. *Epidemiology* **26**, 862–872 (2015).

23. L. L. H. Lau, *et al.*, Viral Shedding and Clinical Illness in Naturally Acquired Influenza Virus Infections. *J. Infect. Dis.* **201**, 1509–1516 (2010).

24. D. Yamin, R. D. Balicer, A. P. Galvani, Cost-effectiveness of influenza vaccination in prior pneumonia patients in Israel. *Vaccine* **32**, 4198–4205 (2014).
